# Diversity and Metabolic Potentials of Subsurface Crustal Microorganisms from the Western Flank of the Mid-Atlantic Ridge

**DOI:** 10.3389/fmicb.2016.00363

**Published:** 2016-03-18

**Authors:** Xinxu Zhang, Xiaoyuan Feng, Fengping Wang

**Affiliations:** ^1^State Key Laboratory of Microbial Metabolism, School of Life Sciences and Biotechnology, Shanghai Jiao Tong UniversityShanghai, China; ^2^State Key Laboratory of Ocean Engineering, School of Naval Architecture, Ocean and Civil Engineering, Shanghai Jiao Tong UniversityShanghai, China

**Keywords:** comparative metagenomics, deep biosphere, geomicrobiology, iron metabolism, oceanic crust

## Abstract

Deep-sea oceanic crust constitutes the largest region of the earth’s surface. Accumulating evidence suggests that unique microbial communities are supported by iron cycling processes, particularly in the young (<10 million-year old), cool (<25°C) subsurface oceanic crust. To test this hypothesis, we investigated the microbial abundance, diversity, and metabolic potentials in the sediment-buried crust from “North Pond” on western flank of the Mid-Atlantic Ridge. Three lithologic units along basement Hole U1383C were found, which typically hosted ∼10^4^ cells cm^-3^ of basaltic rock, with higher cell densities occurring between 115 and 145 m below seafloor. Similar bacterial community structures, which are dominated by Gammaproteobacterial and Sphingobacterial species closely related to iron oxidizers, were detected regardless of variations in sampling depth. The metabolic potentials of the crust microbiota were assayed by metagenomic analysis of two basalt enrichments which showed similar bacterial structure with the original sample. Genes coding for energy metabolism involved in hydrocarbon degradation, dissimilatory nitrate reduction to ammonium, denitrification and hydrogen oxidation were identified. Compared with other marine environments, the metagenomes from the basalt-hosted environments were enriched in pathways for Fe^3+^ uptake, siderophore synthesis and uptake, and Fe transport, suggesting that iron metabolism is an important energy production and conservation mechanism in this system. Overall, we provide evidence that the North Pond crustal biosphere is dominated by unique bacterial groups with the potential for iron-related biogeochemical cycles.

## Introduction

Oceanic crust microbiology has long been ignored and is not well studied due to technical constraints; however, the crust has been assumed to harbor active microorganisms that may significantly contribute to global biogeochemical cycles and weathering of the seafloor landscape (Schrenk et al., 2010; Wang et al., 2013). Several lines of evidence have revealed the presence of microorganisms in this dark, oligotrophic biosphere (Fisk et al., 1998; Cowen et al., 2003; Santelli et al., 2008; Lever et al., 2013); however, some fundamental questions remain, including (1) how much microbial biomass is present in the oceanic crust, (2) where do the microorganisms originate, and (3) what are their metabolic functions.

The recent Integrated Ocean Drilling Program (IODP) expeditions dedicated to microbiology (Expedition 327 Scientists, 2010; Expedition 329 Scientists, 2011; Expedition 330 Scientists, 2011; Expedition 336 Scientists, 2012b) support the investigation of the basalt-hosted oceanic crust and the collection of uncontaminated samples for microscopic and molecular analysis. Previous studies attempted to count cells from seafloor-exposed basalts (Einen et al., 2008; Santelli et al., 2008; Jacobson Meyers et al., 2014), subsurface gabbros (Mason et al., 2010) and crustal fluids (Jungbluth et al., 2013). The results showed that cell densities in the seafloor-exposed crust were between 10^6^ and 10^9^ cells cm^-3^, while those in the subsurface had lower cell densities (<10^5^ cell cm^-3^). Diverse microbial communities from crustal environments have been detected by culture-dependent and -independent techniques spanning a large range of bacterial phyla. For example, Deltaproteobacteria, Firmicutes, Gammaproteobacteria, and Bacteroidetes are present in the flanks of the Juan de Fuca Ridge (JdFR) and the Costa Rica Rift (Nigro et al., 2012; Jungbluth et al., 2013, 2014). Seafloor basaltic glass from the East Pacific Rise (Santelli et al., 2008, 2009) and the Arctic spreading ridges (Lysnes et al., 2004), altered basalts from the Hawaiian Loihi Seamount (Templeton et al., 2005; Santelli et al., 2008; Jacobson Meyers et al., 2014) and the Mid-Atlantic Ridge (Rathsack et al., 2009; Mason et al., 2010) are dominated by Gammaproteobacteria and Alphaproteobacteria. Extracellular enzyme activity tests, functional gene analysis, carbon and sulfur isotopic signatures and laboratory incubations demonstrated the presence of active microorganisms involved in methane- and sulfur-cycling and organic matter transformations (Mason et al., 2010; Lever et al., 2013; Jacobson Meyers et al., 2014; Robador et al., 2015; Supplementary Table S1). However, these studies were restricted to seafloor-exposed basaltic habitats (Templeton et al., 2005; Einen et al., 2008; Santelli et al., 2008), subsurface crustal environments with high temperature basalts (Nigro et al., 2012; Jungbluth et al., 2013), and mantle-type rock (Brazelton et al., 2010; Mason et al., 2010). The microbial life of the young, cool subsurface basalts in ridge flank systems, which represent a more common hydrologically active type of ocean crust (Edwards et al., 2012), has not been characterized yet.

Integrated Ocean Drilling Program Expedition 336 drilled the basaltic basement at “North Pond” (NP), which is located on the western flank of the Mid-Atlantic Ridge (Expedition 336 Scientists, 2012b). Numerous hydrological, geological, and geochemical data have been collected at this site from previous ocean drilling (Becker et al., 2001) and site surveys (Langseth et al., 1992; Picard and Ferdelman, 2011; Ziebis et al., 2012). The data indicated that this area was characterized by vigorous, oxic seawater circulation within the young basaltic crust under a <300 m sedimentary pile (Expedition 336 Scientists, 2012b; Ziebis et al., 2012). NP was thus suggested as a model system for studying subsurface basalt-hosted microorganisms (Bach and Edwards, 2003; Edwards et al., 2012). Modeling approaches suggested the presence of significant biotic oxygen consumption in the upper oceanic crust (Orcutt et al., 2013b). Collectively, the few explorations of the NP crustal biosphere suggested the existence of a unique subsurface biosphere in this system, probably supported by energy produced through iron cycling processes (Thorseth et al., 2001; Bach and Edwards, 2003; Edwards et al., 2012; Scott et al., 2015). To test these assumptions, we analyzed the microbial abundance, diversity and metagenomic properties of basalts collected from basement Hole U1383C with a penetration depth of 324 m below seafloor (mbsf). This is the first study of the vertical distribution of microbial communities in the cool, oxic subsurface oceanic crust, and it provides direct evidence to support the hypothesis that the NP crust hosts a unique biosphere with iron metabolizing potential.

## Materials and Methods

### Sample Collection and Incubation

Basaltic basement samples were collected from North Pond on the western flank of the Mid-Atlantic Ridge during IODP Expedition 336 (Supplementary Figure S1). The methods for collecting and processing the samples, including quality and contamination assessments, are detailed in the Supplementary Material and elsewhere (Expedition 336 Scientists, 2012a). Briefly, samples were checked for the presence of fluorescent microspheres used during coring according to the protocol of Smith et al. (2000), and a drilling mud sample was collected to assess the possibility of contamination from drilling. Only interior pieces of rock were selected for microbiological study to avoid potential drilling mud contamination as recommended elsewhere (Expedition 330 Scientists, 2012; Lever et al., 2013). Samples used for cell enumeration were fixed with paraformaldehyde directly on shipboard. In total, 23 basalt samples that passed the contamination tests from Hole U1383C were used for cell enumeration, seven basalt samples were used for 16S rRNA gene sequencing, and one basalt sample was used for enrichment culturing. Properties of these samples, including porosity and P_2_O_5_ content of the basaltic rocks, are described in more detail elsewhere (Expedition 336 Scientists, 2012c) and the Supplementary Material.

A series of enrichments with the addition of a carbon substrate (sodium bicarbonate, sodium acetate or methane) and/or a nitrogen substrate (ammonium chloride or sodium nitrate) were set up to stimulate the growth of the microorganisms. Briefly, 2 cm^3^ of each rock sample was mixed with 5 mL of 0.22 μm-mesh filtered seawater (collected on site at a water depth of ∼100 m) in a sterile 18 mm × 150 mm glass tube, and each substrate was added at a final concentration of 1.5–3 mM or 20% [vol/vol] headspace. The tube was capped with a butyl rubber stopper and an aluminum seal with filter-sterilized air in the headspace. After 6 months of incubation at 10°C in the dark, 2 mL of thoroughly mixed slurry, which contained suspended rock particles and seawater, was transferred and preserved in an equal volume of 1x phosphate buffered saline (PBS)/ethanol at –20°C until analysis. A parallel incubation was conducted with a double autoclaved basalt sample and incubated in the same conditions as the sterile control. The latter control showed no amplification of bacterial/archaeal 16S rRNA genes and no microbial cells by epi-fluorescence microscopy, which indicated that in this study, 0.22-μm mesh was appropriate for seawater media sterilization, a result similar to those in previous reports (Meron et al., 2011; Zeng and Chisholm, 2012; Reveillaud et al., 2014).

### Cell Enumeration

Cell enumeration was performed after cell extraction, which was conducted following a protocol of Kallmeyer et al. (2008) with a few modifications. Each reagent used before the cell enumeration steps was filter sterilized through a 0.22 μm-mesh membrane filter (Millipore, Billerica, MA, USA). The cell enumeration blank was performed without a sample and processed with the same steps as the basalt samples. Each sample was extracted and counted in triplicate. An average of 200 fields of view was counted for each membrane. The area of each field of view was set at 10,000 μm^2^, and the detection limit was ∼10^3^ cells cm^-3^ for a 95% probability of detecting at least 1 cell as described by Kallmeyer et al. (2008). Details of the method are provided in the Supplementary Material.

### DNA Extraction and 16S rRNA Gene Sequencing

DNA was extracted using a FastDNA^TM^ SPIN Kit for Soil (MP Biomedicals, Santa Ana, CA, USA) according to the manufacturer’s instructions with a few modifications. Approximately 0.5 g of rock was ground into powder with a double flame-sterilized mortar and pestle. To the powder, 5 μg of clean, UV-irradiated poly-dIdC (Sigma–Aldrich, St. Louis, MO, USA) was added to increase the yield of DNA for low-biomass rock samples according to Barton et al. (2006). Three parallel extractions were performed for each sample, and the DNA extracts were pooled for subsequent PCR amplification. All procedures were performed in a laminar flow hood with 70% ethanol decontaminated pipettes, autoclaved filter pipette tips and UV-irradiated reagents. The V4 region of the bacterial 16S rRNA gene was amplified using the multi-tag primers 520F and 802R (Supplementary Table S2), and ∼240 bp amplicons were generated. The PCR program involved an initial denaturation step at 95°C for 10 min, followed by 30 cycles of denaturation at 95°C for 45 s, annealing at 55°C for 1 min, and extension at 72°C for 1 min, with a final extension at 72°C for 5 min. Three parallel PCR amplifications were performed for each sample and pooled for subsequent sequencing. The DNA blank extraction was performed without a sample and processed with the same DNA extraction and PCR amplification kits as the basalt samples. DNA extraction and PCR amplification were considered free of contamination if no target PCR band of approximately 240 bp was seen on an agarose gel for the blank DNA extraction and the PCR negative control.

The 16S rRNA gene amplicons containing unique 8-mer barcodes used for each sample were pooled at equal concentrations, and sequenced on an Illumina MiSeq platform using 2 × 250 bp cycles and the MiSeq Reagent Kit v2 (500 cycle, Illumina, USA) according to manufacturer’s instructions. Raw reads were removed if they contained a 50 bp continuous fragment with an average quality score less than 30 and/or any ambiguities. Filtered reads were merged together using FLASH (Magoč and Salzberg, 2011; Version 1.2.6). Merged sequences were removed if they contained more than six identical bases occurred continuously and/or any ambiguities, or the sequence length was <200 bp. Clean sequences were demultiplexed using the QIIME software pipeline (Caporaso et al., 2010b; Version 1.9.0) with a mapping file containing the sample ID, barcode and primer sequence. Three sets of archaeal primers were tested using conditions described elsewhere (see Supplementary Table S2), but no positive PCR bands were observed.

### Bacterial Community Composition and Phylogenetic Analysis

Sequences were aligned with PyNAST (Caporaso et al., 2010a; Version 1.2.2) and clustered into operational taxonomic units (OTUs) at 97% sequence similarity cutoff using usearch61 (Edgar, 2010) with default parameters in the QIIME software pipeline (Caporaso et al., 2010b; Version 1.9.0). OTUs were assigned to taxa based on the Greengenes database (DeSantis et al., 2006; Version gg_13_5). Chimeric sequences were detected with the UCHIME program (Edgar et al., 2011; Version 4.2) using default parameters, and they were removed from further analysis. Cluster analysis of the microbial community structure was performed in *R* based on a Bray–Curtis matrix using average linkage (R Core Team, 2015; Version 3.2.0). Phylogenetic trees were constructed in QIIME using the FastTree method, and the Shimodaira–Hasegawa test was used to estimate the reliability of each branch with 1000 resamples (Price et al., 2010; Version 2.1.3). Sequences covering the V4 region of the 16S rRNA gene from type species (downloaded from GenBank) and species from the crustal environments of the Mid-Atlantic Ridge (Rathsack et al., 2009; Mason et al., 2010), the JdFR (flanks; Mason et al., 2007, 2009; Orcutt et al., 2011a; Smith et al., 2011; Jungbluth et al., 2013, 2014), the Costa Rica Rift flank (Nigro et al., 2012), the East Pacific Rise (Mason et al., 2007; Santelli et al., 2008, 2009), the Hawaiian Seamounts (Templeton et al., 2005; Santelli et al., 2008; Edwards et al., 2011), and the Takuyo-Daigo Seamount (Nitahara et al., 2011) with sequence similarity to the North Pond sequences were included in the trees. FigTree^1^ was used to modify the phylogenetic trees. In the phylogenetic trees, a representative sequence for each OTU (the most abundant one) is shown rather than all the sequences due to the high sequence number. For the Venn diagram, sequences were rarefied to an even depth (7952 reads; **Table 1**) by random sampling using QIIME. Sample 5R-1B and 20R-2C were excluded from the diagram due to (1) the limit of the Venn diagram presentation and (2) the similarity of their bacterial community compositions to those of 2R-2E and 6R-1A, respectively. The Venn diagram was created with a web tool provided by the Bioinformatics and Systems Biology of Gent^2^

**Table 1:** Number of high quality bacterial 16S rRNA gene sequences used in this study.

Sample ID	Depth (mbsf)	Description^†^	Number of sequences	OTUs (97% cutoff)
2R-2E	72	Aphyric basalt, highly altered	7952	562
5R-1B	97	Aphyric basalt, moderately altered	10472	646
6R-1A	105	Aphyric basalt, slight alteration	9936	548
9R-2C	137	Phyric basalt, extensive alteration	9922	691
10R-1B	145	Phyric basalt, highly altered	10366	754
20R-2C	221	Aphyric basalt, extremely hard	16122	734
30R-1A	304	Aphyric basalt, fractured	16146	596
10R-1B-1	145	Bicarbonate + Ammonium	16283	714
10R-1B-2	145	Bicarbonate + Nitrate	20477	803


*^†^Described by Expedition 336 Scientists (2012c).*

To assess potential contaminating sequences from the reagent kits, a low-biomass contaminant database was constructed using sequences from Tanner et al. (1998), Kulakov et al. (2002), and Barton et al. (2006). All of the OTUs that were assigned to the same taxa with the contaminating sequences were used to construct phylogenetic trees using the same method as described above. A representative set of sequences for each OTU (the most abundant one) were used due to the high sequence number. If an OTU was closely related to sequences of the low-biomass contaminant database, it was further compared with the contaminating sequence using ClustalW Alignment (Thompson et al., 1994; Version ClustalW2) to give a sequence similarity value.

### Metagenomic Sequencing and Analysis

Metagenomic sequencing of the original basalt from 145 mbsf (10R-1B) and its two enrichments (10R-1B-1, sodium bicarbonate + ammonium chloride; 10R-1B-2, sodium bicar bonate + sodium nitrate) which showed the best stimulation of cell growth were performed. To obtain sufficient amounts of DNA for sequencing, whole genome amplification of the total DNA was performed with REPLI-g Mini Kits (Qiagen, Hilden, Germany) following the manufacturer’s protocol. Amplified DNA was further purified using QIAamp DNA Mini Kit (Qiagen, Hilden, Germany) according to the manufacturer’s recommendations. The amplification was conducted in five separate reactions, and they were pooled for subsequent sequencing to reduce amplification biases. Parallel blank controls, including sampling, DNA extraction and amplification controls, were performed with 0.22 μm-mesh membrane filtered Milli-Q water (18.2 MΩ; Millipore, Billerica, MA, USA). Sequencing was performed on HiSeq 2000 platform (Illumina, San Diego, CA, USA) using 2 × 100 bp pair-end technology. Due to the challenging nature of sample retrieval and DNA recovery, replication of the metagenomes was not possible. Raw reads that passed the quality control were assembled into contigs by SOAPdenovo2 (Luo et al., 2012; Version 2.0.4). Gene prediction, annotation, and taxonomic assignments were performed as described previously (He et al., 2013b; Wang et al., 2014). Statistical significance between samples was analyzed by SPSS 13.0 software using the Mann–Whitney *U* test.

### Comparing the Relative Abundances of Iron-Related Pathways

Metagenomes were subject to tBLASTn (Altschul et al., 1990) searches against an iron uptake and transport-related gene database, which was collected from Hopkinson and Barbeau (2012) and Toulza et al. (2012). Matched genes, with a maximum *e*-value of 10^-5^ and minimum identity of 30%, were assigned to specific pathways. The abundance of each pathway is the total count of hits that matched the database, except for the NP and JdFR flank fluid metagenomes, which were adjusted by using the average read depth of the corresponding contig as described elsewhere (He et al., 2013a). The relative abundance of each iron-related pathway among the metagenomes was normalized by estimating the number of sequenced genome equivalents with a set of 30 essential single-copy genes (Nayfach and Pollard, 2015; MicrobeCensus Version 1.0.4). The statistical significance between samples was analyzed by SPSS 13.0 software using the Mann–Whitney *U* test.

### Accession Number

All sequence data have been deposited in the National Center for Biotechnology Information (NCBI) Sequence Read Archive under the accession number SRP042264.

## Results

### Microbial Abundance

**FIGURE 1:**
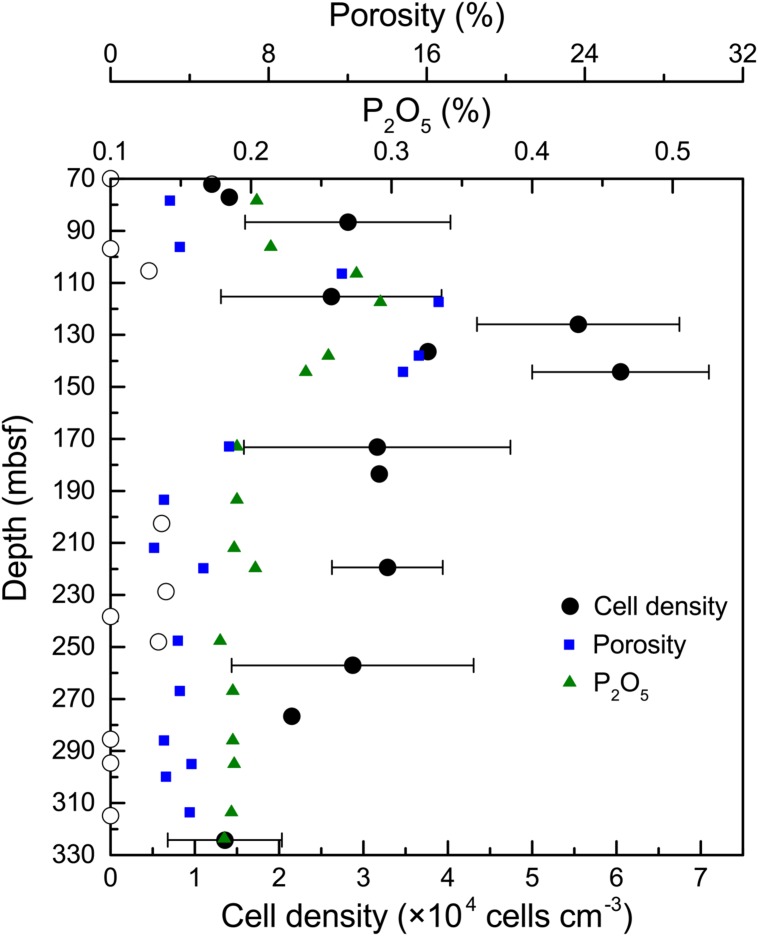
**Microbial abundance in basalt samples from Hole U1383C.** The cell counting data are averages of three determinations. The error bars indicate the standard deviations of the cell counts. Open circles indicate suspicious values near or below the detection limit (∼10^3^ cells cm^-3^). Porosity and P_2_O_5_ content of the basalts are from Expedition 336 Scientists (2012c).

In general, the microbial abundances in the basalts at basement Hole U1383C were between <1.0 × 10^3^ and 6.1 × 10^4^ cells cm^-3^ (**Figure 1**). The highest densities of microorganisms in the basalt occurred between 115 and 145 mbsf, and the biomass was distributed heterogeneously above or below detection at the other depths, with those above detection being ∼1–3 × 10^4^ cells cm^-3^. Samples from the top and the bottom sections gave similar, low values (for example 72 mbsf and 324 mbsf, 1.2 × 10^4^ – 1.4 × 10^4^ cells cm^-3^, respectively), although some samples were near or below the detection limit (∼10^3^ cells cm^-3^).

### Microbial Community Structure

Seven basalt samples from 72 to 304 mbsf were used for microbial community structure characterization (**Table 1**). Across all the basalt samples, no archaeal 16S rRNA gene bands were amplified using different archaeal primer sets (see the section “Materials and Methods”). This indicated a low abundance of archaea, if present, which was consistent with previous studies in other oceanic crust environments (Einen et al., 2008; Santelli et al., 2008; Mason et al., 2010). The number of high quality bacterial 16S rRNA gene sequences obtained by high-throughput sequencing were between 7,952 and 16,146, resulting in more than 548 OTUs at 97% SSU rRNA gene sequence similarity. Alpha-diversity analysis showed that the rarefaction curves for all of the samples did not plateau at this sequencing depth (Supplementary Figure S2A), but the Shannon Diversity indices were stable (Supplementary Figure S2B). This indicated that major bacterial communities had largely been covered, although rare new phylotypes could still appear upon further sequencing.

To assess the potential contamination of rocks from the drilling mud during sampling (Lever et al., 2006) and the potential contaminating sequences from commercial kits used for DNA extraction (Salter et al., 2014), we conducted a comparative taxonomic/phylogenetic analysis of the 16S rRNA gene sequences from this study and those from the procedural controls (see the section “Materials and Methods” for more details). First, no sequences of common surface seawater bacteria (e.g., Cyanobacteria) were recovered in the sequence library, indicating that surface seawater derived drilling fluids did not contaminate the rocks. Second, comparative taxonomic analysis of the sequences from this study with those from the drilling mud sample revealed a distinct separation of rock-hosted microorganisms from drilling mud influence (J. Meyer, J. Huber, personal communication). Third, a comparison of the phylogenetic analysis of the sequence library with a known low-biomass contaminant database (Tanner et al., 1998; Kulakov et al., 2002; Barton et al., 2006) was conducted. As suggested by Barton et al. (2006), any sequences that demonstrated >98% sequence similarities to the contaminant database were flagged as possible contaminants. Six OTUs (representing <11.8% of the total quality-screened sequences) were identified as having 98% or greater sequence similarity to known contaminating sequences, including those from *Acinetobacter*, *Bradyrhizobium*, *Curvibacter*, *Ralstonia*, *Sphingomonas*, and *Stenotrophomonas* (Supplementary Figure S3; Supplementary Table S3). However, cultures from the genera *Ralstonia*, *Sphingomonas*, and *Comamonas* (*Acidovorax*) have recently been reported from basalts collected from this same expedition (Hirayama et al., 2015), and their 16S rRNA genes showed high sequence similarity with those from this study, indicating that removal of these flagged sequences from the library may not be warranted. Thus, sequences identified as possible sequence contaminants are retained in the library but are highlighted in the overall description of the community structure.

**FIGURE 2:**
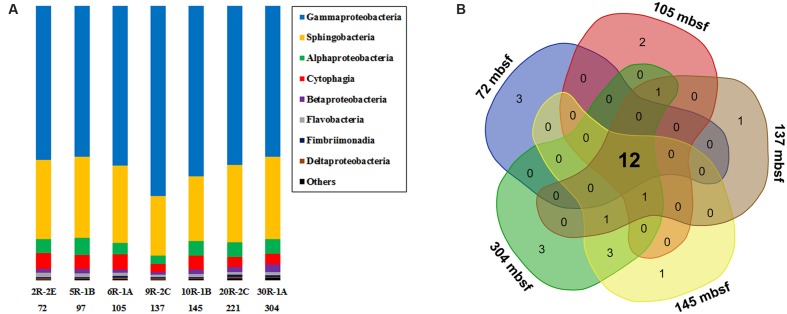
**(A)** The bacterial community composition of basalt samples (at class level). Colored bars indicate the percentage of the designated group within each sample. Only those with >0.5% class abundance are listed. The remaining sequences are grouped to “Others.” The number indicates sample depth, mbsf. **(B)** A Venn diagram showing the distribution of shared bacterial groups (at class level) in samples from 72, 105, 137, 145, and 304 mbsf.

In general, Gammaproteobacteria dominated the bacterial community, ranging from 54.9 to 69.3% at different depth intervals (**Figure 2A**). The greatest abundance of Gammaproteobacteria occurred at 137 mbsf, whereas samples from the top and bottom sections gave similar but lower values. The second most abundant group was Sphingobacteria, showing a relative abundance between 21.8 and 30.0%. Alphaproteobacteria and Cytophagia were generally less than 10% in relative abundance. Other groups with >0.5% relative abundance were Betaproteobacteria, Flavobacteria, Fimbriimonadia, and Deltaproteobacteria. As the microbial biomass in the rocks was low (**Figure 1**), it was not yet clear if and how much the PCR primers preference, and the number of 16S rRNA gene copies in different microbial cells may influence the 16S rRNA gene abundance in the samples (Vetrovsky and Baldrian, 2013). Therefore, we did not tend to compare the differences in the percentages of bacterial 16S rRNA gene sequences in different samples, as the abundance of the bacterial groups were likely under- or overrepresented through the present PCR sequencing methods. We then listed the microbial groups with >1% abundance as major groups in the samples. The microbial community structures shared much in common at different depth intervals, as shown by a Venn diagram (**Figure 2B**). The overlapping groups covered more than 99.8% of the total bacterial community within each sample (Supplementary Table S4). At the genus level, the major groups in the basalts included *Marinobacter*, *Sediminibacterium*, *Comamonas*, *Marivirga*, *Alcanivorax*, *Alteromonas*, *Pseudomonas*, *Methylophaga*, *Oleibacter*, and *Halomonas* (Supplementary Figure S4). The overlapping groups at the genus level covered ∼99% of the total sequences within each sample, regardless of the sample types and depths. Cluster analysis revealed that the samples from 72, 97, 105, and 221 mbsf grouped together based on a Bray–Curtis matrix. Samples from 145 and 304 mbsf formed monophyletic lineages separately, and they were adjacent to the sample from 221 mbsf. The basalt at 137 mbsf formed a distant lineage; however, the community structure was similar to those of other samples, showing a maximum dissimilarity of 0.18 with the sample at 304 mbsf (Supplementary Figure S4).

Spearman’s correlation analysis was performed to identify the distribution of specific crustal bacterial species that was affected by depth. Correlations between OTU abundance and depth were considered significant when *P* < 0.05 and |*R*| > 0.6. In general, 75 OTUs showed positive correlations with depth and 24 OTUs showed the opposite trend, covering a large species diversity within major groups (Supplementary Table S5).

### Phylogeny

**FIGURE 3:**
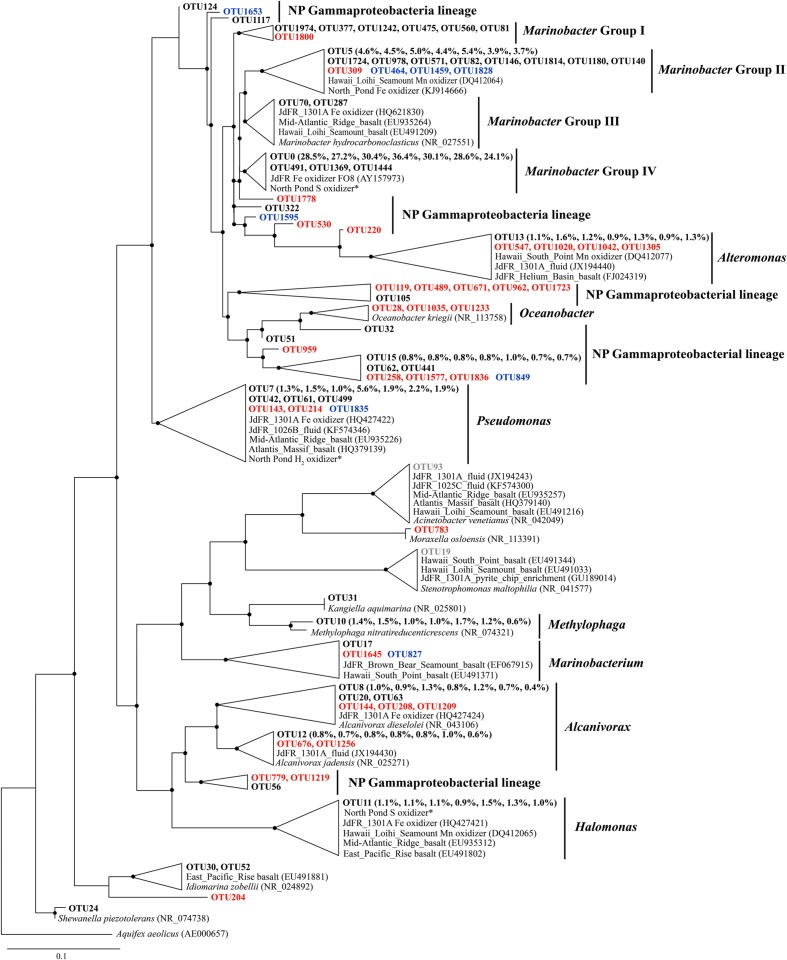
**The Phylogenetic tree of Gammaproteobacterial-related OTUs from seven basalt samples.** Sequences recovered in this study are highlighted in bold font. The numbers in parentheses indicate percentage of the designated reads clustered in 97% cutoff OTUs in the order 72, 97, 105, 137, 145, 221, and 304 mbsf. Sequences in red are positively correlated with depth, those in blue are negatively correlated with depth, and those in gray are potential contaminants. ^∗^ indicates sequences from Hirayama et al. (2015). Only those with >70 local support values are shown as filled circles at each branch. Only those with >0.1% OTU abundance within each sample and a representative sequence for each OTU (the most abundant one) are used due to the large number of sequences. The 16S rRNA gene of *Aquifex aeolicus* (AE000657) is used as the outgroup. The scale bar indicates 0.1 nucleotide substitutions per site.

Phylogenetic analysis revealed a single cluster that was closely related to a cultured neutrophilic Fe-oxidizing bacterium isolated from weathering deposits of the JdFR axis (Edwards et al., 2003; designated *Marinobacter* Group IV; **Figure 3**). The most diverse subcluster within the genus *Marinobacter* was *Marinobacter* Group II in the Gammaproteobacteria. Sequences related to this group included an iron oxidizing bacterium isolated from 304 mbsf (30R-1A) of U1383C (GenBank accession number KJ914666; unpublished), and a manganese oxidizing bacterium from the basalts of the Hawaiian Loihi Seamount (Templeton et al., 2005). *Marinobacter* Group III is characterized as a common lineage in marine basaltic habitats, including the JdFR flanks, the Mid-Atlantic Ridge and the Hawaiian Loihi Seamount. Some cultured strains of this group have been demonstrated to oxidize iron under neutrophilic conditions (Smith et al., 2011). One of these strains was also a known hydrocarbon degrader (Huu et al., 1999). Seven OTUs from Group I of *Marinobacter* formed a monophyletic lineage that was distinct from any cultured *Marinobacter* species, with the best BLAST hit (identity < 97%) to a recently identified *Marinobacter* species from the Sea of Japan (Ng et al., 2014). OTUs that were assigned to *Pseudomonas*, *Alcanivorax*, *Halomonas*, and *Alteromonas* were closely related to Fe/Mn oxidizers isolated from subsurface crustal fluids of the JdFR flank and/or seafloor basalts near Hawaii, and some of them also clustered with environmental gene clones from the Mid-Atlantic Ridge (Atlantis Massif) and/or the East Pacific Rise. In addition, OTUs that may participate in iron reduction and iron assimilation were detected and showed close relationships to known iron reducers, including *Alteromonas* (Lovley et al., 1989), *Shewanella* (Wu et al., 2013), and siderophore producing *Pseudomonas* (Paulsen et al., 2005). A single OTU related to *Methylophaga* was unique to NP basalts and indicated the potential to use one-carbon compounds (Villeneuve et al., 2013).

**FIGURE 4:**
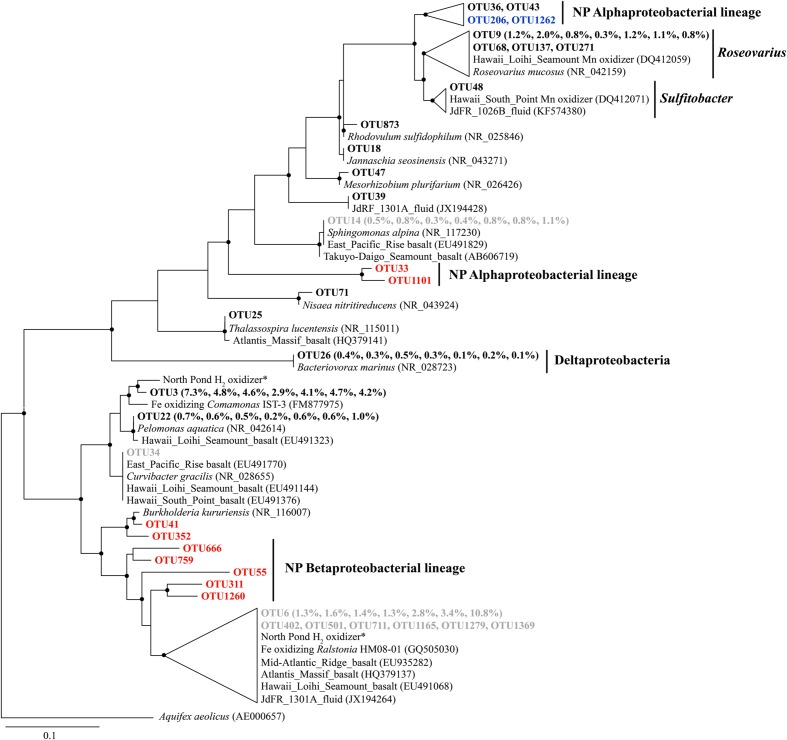
**The phylogenetic tree of Beta-, Alpha-, and Delta-proteobacterial-related OTUs from seven basalt samples.** Sequences recovered in this study are highlighted in bold font. The numbers in parentheses indicate the percentage of the designated reads clustered in 97% cutoff OTUs in the order 72, 97, 105, 137, 145, 221, and 304 mbsf. Sequences in red are positively correlated with depth, those in blue are negatively correlated with depth, and those in gray are potential contaminants. ^∗^ indicates sequences from Hirayama et al. (2015). Only those with >70 local support values are shown as filled circles at each branch. Only those with >0.1% OTU abundance within each sample and a representative sequence for each OTU (the most abundant one) are used due to the large number of sequences. The 16S rRNA gene of *Aquifex aeolicus* (AE000657) is used as the outgroup. The scale bar indicates 0.1 nucleotide substitutions per site.

Some Alphaproteobacterial OTUs from *Roseovarius* and *Sulfitobacter* clustered with Mn oxidizers recovered from the Loihi Seamount and South Point near Hawaii (**Figure 4**), although they were generally present in low relative abundance (<1%). The *Bacteriovorax*-related sequences were the only identified Deltaproteobacterial OTU, and they shared 100% sequence similarity with *Bacteriovorax marinus*, a marine bacterium that preys upon Gram-negative bacteria (Baer et al., 2004).

**FIGURE 5:**
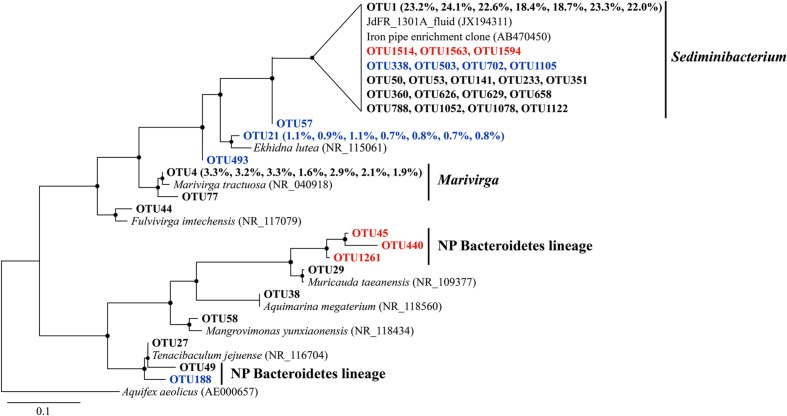
**The phylogenetic tree of Bacteroidetes-related OTUs from seven basalt samples.** Sequences recovered in this study are highlighted in bold font. The numbers in parentheses indicate the percentage of the designated reads clustered in 97% cutoff OTUs in the order 72, 97, 105, 137, 145, 221, and 304 mbsf. Sequences in red are positively correlated with depth, those in blue are negatively correlated with depth. Only those with >70 local support values are shown as filled circles at each branch. Only those with >0.1% OTU abundance within each sample, and a representative sequence for each OTU (the most abundant one) is used due to the large number of sequences. The 16S rRNA gene of *Aquifex aeolicus* (AE000657) is used as the outgroup. The scale bar indicates 0.1 nucleotide substitutions per site.

Sequences that were assigned to *Sediminibacterium* were closely related to environmental clones recovered from crustal fluids of the JdFR flank and an enrichment clone from iron pipes of a water distribution system (Wang et al., 2012) (**Figure 5**). Other abundant OTUs were generally related to aerobic heterotrophs, including *Marivirga* and *Ekhidna*, which were isolated from marine environments and characterized by gliding motility (Lewin and Lounsbery, 1969; Alain et al., 2010).

### Metabolic Potential

Metagenomic sequencing of the original basalt from 145 mbsf (10R-1B) and its two enrichments were performed as described in the Section “Materials and Methods.” Unfortunately, the assembly of the original basalt sample resulted in only ∼1 Mbp, and, although multiple assembly software applications were tested [including SOAPdenovo2 (Luo et al., 2012; Version 2.0.4), Velvet (Zerbino and Birney, 2008; Version 1.2.09), ABySS (Simpson et al., 2009; Version 1.5.1), and SPAdes (Bankevich et al., 2012; Version 3.5.0)], it had to be excluded from further analysis. In general, more than 99.3% of the total number of sequences were assigned to bacteria [rules for taxonomic assignment were detailed by He et al. (2013b)]. The archaeal sequences were present at less than 0.2% in the two metagenomes (Supplementary Table S6), which was consistent with previous results. The taxonomic composition of these two basalt enrichments based on 16S rRNA gene amplification revealed that they shared much similarity with those in the original rock (Supplementary Figure S4). Integrated study of these two metagenomes may shed some light on the potential functions of the indigenous microbiome in the basalt, although they were not representative of the *in situ* environment.

The proteins predicted from the metagenomes of the NP basalt enrichments were classified into functional gene categories based on the Subsystems database (Overbeek et al., 2005). Hierarchical clustering was conducted by comparing metagenomes from diverse environments, including two subsurface crustal fluids from the JdFR flanks (GOLD ID: Gs0090290; Reddy et al., 2015), a carbonate chimney from the Lost City (Brazelton and Baross, 2009), a sulfide chimney from the JdFR (Xie et al., 2011), an oil-immersed chimney from the Guaymas Basin (He et al., 2013b), an iron oxide mat from the Loihi Seamount (Singer et al., 2013), three water samples from the Sargasso Sea (MG-RAST ID: 4539502.3, 4539504.3, 4539507.3; Meyer et al., 2008), two subseafloor sediments from the Peru Margin (Biddle et al., 2008) and two pyrite sediments from California acid mine drainage (Tyson et al., 2004; Lo et al., 2007).

The metagenomes of the NP basalt enrichments clustered closely with samples from the subsurface crustal fluids of the JdFR flanks, the iron mat from the Loihi Seamount and the Lost City carbonate chimney (Supplementary Figure S5). When we considered those samples as Group 1 and the rest of the samples as Group 2, Group 1 was significantly enriched in functional categories, including flagellar motility, fatty acids, branch-chain amino acids, siderophores (*P* < 0.05), and it was depleted in ATP synthases, quorum sensing, and biofilm formation (*P* < 0.05). Variability in these functional categories appeared to drive this clustering. In addition, the two metagenomes from the NP basalts were enriched in functional categories, including resistance to antibiotics and toxic compounds, fermentation, peripheral pathways for catabolism of aromatic compounds, phospholipids, osmotic stress and tricarboxylate transporter (*P* < 0.05) compared with the rest of the metagenomes. The metagenomes of two subsurface pyrite sediments from California acid mine drainage were adjacent to NP enrichments, indicating their microbial functional similarity with basalt-hosted environments.

To determine the metabolic potentials of the microorganisms in NP basalts involved in carbon, nitrogen, hydrogen, and iron processes, key genes were searched in the assembled contigs. Genes, including those coding for alkane monooxygenase, cytochrome P450, flavin-binding monooxygenase and catechol-2,3-dioxygenase were identified (**Table 2**), suggesting that the microorganisms could degrade hydrocarbons. Carbon fixation via the reductive TCA cycle and the reductive acetyl-CoA cycle (Hugler and Sievert, 2011) were identified but were incomplete; however, key genes, including carbon monoxide dehydrogenase were present in both metagenomes. RuBisCO genes of the Calvin–Benson–Bassham cycle were not detected. In nitrogen metabolism, the complete pathways of dissimilatory nitrate reduction to ammonium (DNRA) and denitrification were both present in the two metagenomes (**Table 2**). However, genes for ammonia oxidation, anaerobic ammonium oxidation (Anammox) and nitrogen fixation pathways were absent. Other genes in the assimilatory nitrate reduction (nitrate reductase, NasA) and ammonia assimilation pathways (glutamine synthetase and glutamate synthase) were identified. Three [NiFe]-hydrogenase genes were identified in the metagenome 10R1B-1 (**Table 2**), indicating that cells may gain energy by the oxidation of hydrogen gas (H_2_).

**Table 2:** Key genes involved in carbon, nitrogen and hydrogen metabolism.

Pathway	Enzyme	Best hit Contig ID		Best Blastp hit organism	Similarity (%)
				
		10R1B-1	10R1B-2		
Dissimilatory nitrate reduction to ammonium	Nitrate reductase alpha subunit, NarG	NP-10R-1B-1_contig4398	NP-10R-1B-2_contig245	*Marinobacter manganoxydans* MnI7-9	99
	Nitrite reductase [NAD(P)H] large subunit, NirB	NP-10R-1B-1_contig29	NP-10R-1B-2_contig1171	*Marinobacter manganoxydans* MnI7-9	99
Denitrification	Nitrite reductase (NO-forming), NirK	NP-10R-1B-1_contig5784	NP-10R-1B-2_contig5271	*Alcanivorax dieselolei* B5	99
	Nitric oxide reductase subunit B, NorB	NP-10R-1B-1_contig2504	NP-10R-1B-2_contig240	*Marinobacter hydrocarbonoclasticus * ATCC 49840	95
	Nitrous oxide reductase, NosZ	NP-10R-1B-1_contig7092	NP-10R-1B-2_contig6914	*Marinobacter manganoxydans* MnI7-9	99
Nitrogen assimilation	Assimilatory nitrate reductase, NasA	NP-10R-1B-1_contig28	NP-10R-1B-2_contig1171	*Marinobacter manganoxydans* MnI7-9	99
	Glutamine synthetase, GlnA	NP-10R-1B-1_contig1716	NP-10R-1B-2_contig1000	*Marinobacter algicola* DG893	99
	Glutamate synthase alpha subunit, GltB	NP-10R-1B-1_contig3559	NP-10R-1B-2_contig709	*Marinobacter algicola* DG893	99
H_2_ oxidation	[NiFe]-hydrogenase large subunit	NP-10R-1B-1_contig3091	ND	Gamma proteobacterium HIMB30	80
Hydrocarbon degradation	Alkane monooxygenase, AlkB	NP-10R-1B-1_contig7840	NP-10R-1B-2_contig151	*Marinobacter manganoxydans* MnI7-9	99
		NP-10R-1B-1_contig3498	NP-10R-1B-2_contig4917	*Alcanivorax dieselolei* B5	99
	Cytochrome P450	NP-10R-1B-1_contig1835	NP-10R-1B-2_contig1725	*Alcanivorax hongdengensis* A-11-3	95
		NP-10R-1B-1_contig10204	NP-10R-1B-2_contig1585	*Marinobacter manganoxydans* MnI7-9	99
	Flavin-binding monooxygenase, AlmA	NP-10R-1B-1_contig909	NP-10R-1B-2_contig4078	*Alcanivorax dieselolei* B5	99
	Catechol-2,3-dioxygenase	NP-10R-1B-1_contig7526	NP-10R-1B-2_contig859	*Marinobacter algicola* DG893	97


*ND, not detected.*

In particular, a comparison of the relative gene abundances for cellular iron uptake and transport pathways within metagenomes from diverse marine environments and a subsurface pyrite-hosted acid mine drainage was conducted by using a database collected from Hopkinson and Barbeau (2012) and Toulza et al. (2012). The results showed that genes for Fe^3+^ uptake, siderophore synthesis and uptake, and unspecified Fe transport were significantly enriched in the basalt-hosted environments (Loihi Seamount and NP) and the deep-sea hydrothermal chimneys (JdFR, Guaymas Basin and Lost City), with *p*-values < 0.05 (Supplementary Figure S6). Cluster analysis further revealed that these samples grouped together (except for those from the Guaymas Basin) and were distinct from the metagenomes of the Peru margin sediments and Sargasso seawater. The subsurface crustal fluids of the JdFR flanks and the pyrite sediments of the California acid mine drainage formed a distant subcluster, which showed a relatively low abundance of iron-related genes. In total, genes involved in Fe^3+^ uptake were always the most abundant in NP basalts, whereas siderophore synthesis and uptake pathways prevailed in an iron oxide microbial mat from the Loihi Seamount.

## Discussion

Using microscopic cell enumeration and molecular techniques, we characterized the microbiota of subsurface basalts from the young, cool oceanic crust at NP. Our results demonstrate that the microbial abundances in the basalts are less than 6.1 × 10^4^ cells cm^-3^, with the microbial communities dominated by Gammaproteobacteria and Sphingobacteria (**Figures 1** and **2**). The microbial abundances of the NP basalts are similar to those of previous measurements of cell densities on subsurface basaltic environments (Fisk et al., 2003; Mason et al., 2010; Jungbluth et al., 2013), which are more than two orders of magnitude lower than those on seafloor-exposed basalts (Einen et al., 2008; Santelli et al., 2008; Jacobson Meyers et al., 2014). Higher cell densities are observed at 115–145 mbsf, indicating potential correlations with *in situ* physical and geochemical characteristics as discussed below. The basalt bacterial community structures at different sampling depths are relatively uniform with numerous bacterial species closely related to cultured iron/manganese oxidizers and environmental clones from various oceanic crustal habitats. Moreover, we identified some bacterial lineages that appear to be localized in NP, indicating that a unique microbial biosphere is hosted in this system. Finally, we suggest that iron-related metabolisms are significant processes in basalt-hosted environments based on comparative metagenomics.

### Distribution of Microbial Abundance and Composition

In contrast to previous studies (e.g., Santelli et al., 2008; Jungbluth et al., 2013), we provide a more detailed characterization of microbial life in the subsurface oceanic crust across a 254 m core. The distribution of microbial abundance in Hole U1383C did not follow the general trend observed in global subsurface marine sedimentary environments, where cell densities decrease logarithmically with increasing sediment depth (Kallmeyer et al., 2012). This trend in sediments was principally controlled by the availability of energy sources, including buried organic matter (D’Hondt et al., 2004; Lipp et al., 2008). Along the core analyzed in our study, higher cell densities were found at depths where higher contents of phosphorus oxide (P_2_O_5_) and porosity occurred in the rock (**Figure 1**). For example, the cell density at 145 mbsf was fivefold higher than in the top sample at 72 mbsf and the bottom sample at 324 mbsf; the content of P_2_O_5_ and the porosity of the basalt at this depth reached 0.3 and 16.6%, respectively, which were among the highest values in the core (Expedition 336 Scientists, 2012c). The co-occurrence of higher cell densities and P_2_O_5_ at 115–145 mbsf suggests that phosphorus is an important nutrient that may control the endolithic microbial biomass. Phosphorus is known to be an essential element for microbial growth. This is consistent with recent observations of low phosphate content in subsurface crustal fluids compared to bottom seawater, suggesting that phosphorus is a limiting nutrient in the subsurface crustal biosphere (Lin et al., 2012). Meanwhile, the co-occurrence of higher cell densities and porosity at these depth intervals could be explained by the fact that (1) high porosity provides more potential surface area available for microbial colonization (Nielsen and Fisk, 2010), and (2) high porosity facilitates higher rates of fluid flow through the basalts (Orcutt et al., 2013b), supplying higher contents of bioavailable nutrients and/or energy from crustal fluids or bottom seawater.

In summary, we see a strong correlation of microbial abundance with basalt P_2_O_5_ and porosity, which suggests that the variation in microbial abundance in subsurface basalts is controlled by geochemical and/or physical changes. However, we cannot preclude the possibility of other parameters *in situ*, due to the challenges of obtaining samples and collecting data as well as the heterogeneous nature of the basalts. For example, because the nitrogen content in the basalt is extremely low (<0.01%; Marty, 1995; Busigny et al., 2005), nitrogen in the crustal fluids, which is the main source of nitrogen, may decrease over time due to consumption to levels that limit microbial growth. Nitrogen limitation is also indicated in altered basalts from Costa Rica Rift (Torsvik et al., 1998).

Considering the large volume of the oceanic crustal habitats (Orcutt et al., 2011b), even the relatively low microbial abundances determined in this study suggest that basalt-hosted microorganisms contribute significantly to global biogeochemical cycles. Extrapolating from this limited dataset of microbial abundance on seafloor-exposed basalts and in subsurface basalts (Supplementary Table S1) to the global volume of this habitat, we estimate that the total microbial biomass could match or exceed the total cells estimated in subseafloor sediments (Kallmeyer et al., 2012), which is consistent with a recent hypothesis (Orcutt et al., 2013a). Modeling approaches based on assumptions of assumed pore space available in the crust suggest that a much higher cell density in the global crust is possible (Heberling et al., 2010), but more investigations of microbial abundance in the crust are needed to constrain these estimates, including microbiological samples from a broader range of crust type, crustal age and permeability conditions.

A similar microbial community was found colonizing the basaltic crust of NP regardless of variations in sampling depth (**Figure 2**). This result was suggested from a previous study of the subsurface gabbroic crust at the Atlantis Massif (Mason et al., 2010), although the study used a low resolution method based on Denaturing Gradient Gel Electrophoresis (DGGE). This suggests that (1) the geochemical redox zonations of the basaltic rock and its surrounding crustal fluids are similar, showing relatively stable ratios of major electron donors (e.g., reduced iron, hydrogen gas and trace amount of dissolved organic carbon) and acceptors (e.g., oxygen and nitrate; Expedition 336 Scientists, 2012c; Ziebis et al., 2012; Orcutt et al., 2013b); (2) the microbial communities are homogeneously distributed within the porous and permeable basaltic crust by the advection dominated crustal fluid flow at NP (Edwards et al., 2012), where the basalts are continuously seeded with microbial cells by crustal fluids; (3) the dominance of these groups showed their potential importance to the dynamics of the basaltic microbial community.

In addition, we retrieved sequences that are not found in other crustal environments and/or without any cultured representatives (the “NP lineage” in the tree), covering diverse bacterial groups (**Figures 3**–**5**). This is mainly owing to the recent high-throughput sequencing technology, which substantially extended our view of microbial diversity and potential metabolic functions inhabiting the cool, oxic subsurface basalt-hosted biosphere. For example, we identified a single lineage of *Marinobacter* Group I, which is distinct from any cultured *Marinobacter* species, indicating a unique lineage localized in the NP basalts. The first detection of *Bacteriovorax*-related sequences provides clues that the predatory lifestyle may be an important survival strategy and contribute to microbial biogeochemical cycles in nutrient-starved environments exemplified by NP. Furthermore, the identification of some OTUs that show positive correlation with sampling depth may suggest that they are characteristic of deep biosphere lineages, especially for those forming monophyletic lineages without cultured representatives.

### Carbon and Energy Metabolism in the Cool, Oxic Subsurface Crust

The exact mechanism of autotrophic fixation of CO_2_ by basalt microorganisms is uncertain due to the lack of key genes involved in the main autotrophic carbon fixation pathways (Hugler and Sievert, 2011). The presence of O_2_ and the δ^13^C-TOC values of basalts (approximately –25‰; Sakata et al., 2015) suggest carbon fixation by the Calvin–Benson–Bassham cycle, an aerobic pathway found in most Fe oxidizers (Emerson et al., 2010; Lever et al., 2013). However, based on the detection of carbon monoxide dehydrogenase, phosphoenolpyruvate (PEP) carboxylase and pyruvate carboxylase, together with the dominance of facultative chemoautotrophs/mixotrophs, we speculate that the subsurface microorganisms at NP use a mixotrophic pathway to assimilate CO_2_ into cellular materials, as proposed elsewhere (Swingley et al., 2007).

The metagenomes from the basalt-hosted environments (e.g., Loihi Seamount, JdFR, and NP) are enriched in genes for Fe^3+^ uptake, siderophore synthesis and uptake, and unspecified Fe transport pathways (Supplementary Figure S6), suggesting that iron-related metabolism could be significant processes supporting life in subsurface basalts from the cool, oxic subsurface crust at NP. Notably, iron oxidation could be an important energy producing process in the basalts. Our diversity analysis showed that some OTUs obtained from NP basalts were closely related to cultured iron oxidizers (**Figures 3**–**5**; Edwards et al., 2003; Blothe and Roden, 2009; Smith et al., 2011; Swanner et al., 2011; Wang et al., 2012; Hirayama et al., 2015). For example, a monophyletic clade, *Marinobacter* Group II, may represent Fe-oxidizing facultative chemoautotrophs based on the phylogenetic data here and elsewhere (Kaye et al., 2011), and an iron-oxidizing *Marinobacter* strain was isolated from 30R-1A basalts at 304 mbsf (GenBank accession number KJ914666), although known iron oxidation genes [e.g., *iro*, *fox, cyc1*, *cyc2*, *cox*, *pio*, and *rus* (as summarized by Ilbert and Bonnefoy, 2013)] and potential candidate genes [e.g., *mtoA* (Liu et al., 2012), *actB* (Refojo et al., 2012), and *cyc2*_PV -1_ (Barco et al., 2015)], were not detected in the metagenomes. If iron oxidation is a dominant process as discussed previously, aqueous Fe^2+^ is transformed to Fe^3+^ at the outer membrane or in the periplasm of microbial cells (Emerson et al., 2010; Ilbert and Bonnefoy, 2013). Abundant genes involved in Fe^3+^ uptake, siderophore uptake and unspecified Fe transport may facilitate the transport of the Fe^3+^ into intracellular materials or the binding to organic complexes, including siderophores (Sandy and Butler, 2009). This may avoid the accumulation of insoluble Fe oxyhydroxide and sulfide minerals on the surface of microbial cells due to the rapid chemical precipitation of Fe^2+^ at circumneutral pH. Excess Fe minerals would cause encrustation and cell death for lack of energy and nutrient availability. Moreover, the relatively high abundances of siderophore synthesis genes in basalt-hosted metagenomes helps to produce more siderophores, which would facilitate the dissolution of solid-phase iron minerals (Kraemer et al., 2005) and, in turn, provide more bioavailable iron for microbial energy-yielding activities. Previous work hypothesized that a significant fraction of the iron oxidation in the young upper oceanic crust (<20 million-year old) could support microbial biomass production in subsurface basalts, given that (1) iron is assumed to be the quantitatively most important bioavailable element in the basalt (8 wt%; Melson et al., 2002), and (2) the kinetic favorability in low-temperature ridge flank systems (Bach and Edwards, 2003; Edwards et al., 2012).

Other energy producing processes may also exist in this system. The detection of a hydrogen oxidation gene in the 10R-1B-1 metagenome (**Table 2**) indicates that microbial life is supported by H_2_ sources generated from water-rock reactions (e.g., serpentinization; McCollom and Bach, 2009). Heterotrophic metabolism is predicted by our metagenome data. Notably, genes involved in hydrocarbon degradation (e.g., alkane monooxygenase, cytochrome P450, flavin-binding monooxygenase, and catechol-2,3-dioxygenase) were identified, indicating that the microorganisms could use hydrocarbons that originated from crustal fluids or bottom seawater (Lin et al., 2012), diffusion from the overlying sediment (D’Hondt et al., 2009), serpentinization reactions (Proskurowski et al., 2008) or even cell lysates (Jover et al., 2014). In addition, heterotrophic metabolism is supported by the enrichment of functional categories, including fermentation and catabolism of aromatic compounds in NP basalts compared to the rest of the metagenomes listed in Supplementary Figure S5. Heterotrophy is also suggested by previous observations of depleted dissolved organic carbon in subsurface crustal fluids compared with the surrounding bottom seawater in the JdFR flank (Lin et al., 2012) and the detection of hydrocarbon degradation genes in the subsurface gabbroic crust at the Atlantis Massif (Mason et al., 2010).

In summary, this study demonstrates that similar microbial communities with relatively low abundance are colonizing the cool, oxic subsurface oceanic crust at NP. Unique microbial communities dominated by Gammaproteobacteria and Sphingobacteria have the potential to play a major role in iron metabolism, which appears to be a significant process in this ecosystem. Although the correlation between microbial abundance and *in situ* physical and geochemical characteristics is indicated in this study, it is still an open question due to the limited data. In addition, the specific contributions of autotrophy versus heterotrophy in the crustal biosphere are still unclear. Ongoing studies at NP (including laboratory incubations, CORK borehole observatory, RNA-based microbial diversity analyses) and future expeditions [e.g., IODP Expedition 357 at the Atlantis Massif (Früh-Green et al., 2015)] may elucidate the variability of microbial abundance and diversity and the balance of autotrophy versus heterotrophy in the oceanic crustal biosphere.

## Author Contributions

FW and XZ designed and performed the experiments, analyzed the data and wrote the manuscript. XF analyzed the data. All authors commented on the manuscript.

## Conflict of Interest Statement

The authors declare that the research was conducted in the absence of any commercial or financial relationships that could be construed as a potential conflict of interest.

## Footnotes

1 http://tree.bio.ed.ac.uk/software/figtree/

2 http://bioinformatics.psb.ugent.be/webtools/Venn/.
