# Supplementary material for: Diversity and Metabolic Potentials of Subsurface Crustal Microorganisms from the Western Flank of the Mid-Atlantic Ridge
Source: Front Microbiol. 2016 Mar 18;7:363. doi: 10.3389/fmicb.2016.00363 (PMC4797314; doi:10.3389/fmicb.2016.00363)
Supplement: Supplementary file 1 [file Data_Sheet_1.DOCX]

***Supplementary Material***

**­­­**

**Diversity and metabolic potentials of subsurface crustal microorganisms from the western flank of the Mid-Atlantic Ridge**

**Xinxu Zhang^1,2^, Xiaoyuan Feng^1^, Fengping Wang^1,2*^**

^1^ State Key Laboratory of Microbial Metabolism, School of Life Sciences and Biotechnology, Shanghai Jiao Tong University, Shanghai, People’s Republic of China.

^2^ State Key Laboratory of Ocean Engineering, School of Naval Architecture, Ocean and Civil Engineering, Shanghai Jiao Tong University, Shanghai, People’s Republic of China.

*** Correspondence:** Fengping Wang, State Key Laboratory of Microbial Metabolism, School of Life Sciences and Biotechnology, Shanghai Jiao Tong University, 800 Dongchuan Road, Shanghai, 200240, People’s Republic of China.

Email: fengpingw@sjtu.edu.cn

**Summary**

The Supplementary Material includes Supplementary Methods, six supplementary figures and six supplementary tables.

**Supplementary Methods**

**Sample collection and contamination tests**

The information is already published in the Methods section of IODP Expedition 336 reports ([Expedition 336 Scientists, 2012a](#_ENREF_6)) and reprinted here.

To examine potential contamination of hard rock and sediment core samples during drilling, slurries of yellow-green fluorescent microspheres (Fluoresbrite Carboxylate Microspheres; Polysciences, Inc., 15700) were sealed in plastic bags and placed inside the core catcher prior to deployment of the core barrel according to standard protocol ([Smith et al., 2000](#_ENREF_26)). Immediately following delivery of basalt cores on deck, only large (>10 cm in length) intact whole-round pieces of rock samples were selected for microbiological study. Combusted aluminum foil or sterile Whirl-Pak bag were used for transport of samples to the microbiology laboratory. Whole-round rock pieces were then transferred to sterile Whirl-Pak bags containing 10 mL of sterile filtered seawater for gentle rinsing and removal of any microspheres and other contaminates. The rinsing process was repeated three times and the rinse was collected into a 15 mL conical vial and stored at 4 °C until processing. Fluorescent microspheres in the rinse were quantified by epi-fluorescence microscopy with a blue filter set according to standard protocol ([Smith et al., 2000](#_ENREF_26)). Both basalt and sediment samples used in this study showed no microsphere after the third wash, indicating that samples passed the contamination test and were generally decontaminated. An exception was that only one microsphere was detected from sample 2R-2E (72 mbsf) in the final wash, which was possibly contaminated during drilling. Next, rocks were transferred to a flame-sterilized rock processing box ([Expedition 327 Scientists, 2011](#_ENREF_4)) and broken into smaller pieces using flame-sterilized chisels and forceps. Only interior pieces of a rock sample was selected for microbiological study as had been shown by multiple studies that the interiors of rock cores are generally free from contamination ([Lever et al., 2006](#_ENREF_15); [Smith et al., 2011](#_ENREF_25); [Expedition 330 Scientists, 2012](#_ENREF_5)). They were further ground into sand-sized fractions with a flame-sterilized steel mortar and pestle and used for subsequent enrichment experiments. All sample handlers wore gloves to avoid contamination during the whole process. To avoid potential contamination and cell death during sample transportation, the enrichment experiments were directly performed on shipboard. Fluorescent microspheres in the basalts and their enrichments were further checked by epi-fluorescence microscopy on delivering to home laboratory. No microsphere was detected from all of the samples (including 2R-2E), which suggested that potential contamination during drilling and experimentation was minimal.

**Porosity and P_2_O_5_ measurements**

Porosity of the basaltic rock was determined using a vacuum water saturator, a dual balance system, and a hexapycnometer. The vacuum pump system was used to keep the hard rock samples completely saturated, and the samples were kept in a vacuum bath of seawater for >24 h. The samples were weighed on the dual balance system (METTLER TOLEDO, Switzerland) to obtain their wet mass. Then the samples were placed in a convection oven at ~105°C for >24 h and their dry mas were measured using the same dual balance system. The samples were placed in the hexapycnometer system to measure dry volume. Porosity (φ) is defined as: φ = V_pw_/(V_d_ + V_pw_), where V_pw_ = pore water volume, V_d_ = dry volume. More details are provided elsewhere ([Expedition 336 Scientists, 2012a](#_ENREF_6)).

For the determination of P_2_O_5_ concentrations in the basaltic rocks, samples were ground to a fine powder in a tungsten carbide SPEX 8000M mixer/mill (SPEX, USA) or, for larger samples, a SPEX 8515 shatter box (SPEX, USA). P_2_O_5_ concentrations were measured using inductively coupled plasma–atomic emission spectroscopy (Teledyne Leeman Labs, USA). Detailed information is provided elsewhere ([Expedition 336 Scientists, 2012a](#_ENREF_6)).

**Cell enumeration**

Cell enumeration was performed after a cell extraction following a protocol applied for low-biomass samples from [Kallmeyer et al. (2008](#_ENREF_12)) with a few modifications. Briefly, 100 μL of preserved slurry was mixed with 500 μL acetate buffer (0.43 M glacial acetic acid, 0.43 M sodium acetate, dissolved in 3.5% NaCl solution) to dissolve carbonates for 2 h. Carbonate-free slurries were centrifuged for 5 min at 3,000 × *g*, and the supernatant was removed and kept for counting. The remaining pellet was then resuspended with 500 μL sodium chloride : formalin solution (3.5% NaCl, 3% formalin), followed by 50 μL detergent mix (100 mM disodium EDTA dihydrate, 100 mM sodium pyrophosphate decahydrate, 1 % Tween 80, in 3.5% NaCl solution) and 50 μL methanol. After vortexing for 2 h, a cushion of 500 μL 50% (wt/vol) Nycodenz^®^ was layered below the slurry with a 12-gauge needle syringe and centrifuged at 1,500 × *g* for 10 min. The supernatant was then transferred to a separate tube, discarding the remaining Nycodenz^®^ layer. The remaining pellet was resuspended in 500 μL sodium chloride solution, 50 μL detergent mix, and 50 μL methanol. The vial was sonicated at 20 W in an ice-water bath for 3×10 s with a 20 s interval. Addition of Nycodenz^®^ followed by centrifugation was repeated as described above and the supernatant was transferred to a separate tube. The supernatants were filtered through a 0.22-μm mesh GTBP membrane (Millipore) and stained with SYBR^®^ Green I solution (1:40 vol/vol SYBR^®^ Green I in 1×Tris-EDTA buffer) for 20 min. The stain solution was removed, the filter was placed onto a glass slide, and 25 μL 10% glycerine was added as an antifade agent. Cells were counted at 1000× magnification using an epi-fluorescence microscope (Nikon, ECLIPSE 90i, Japan) with a blue filter set.


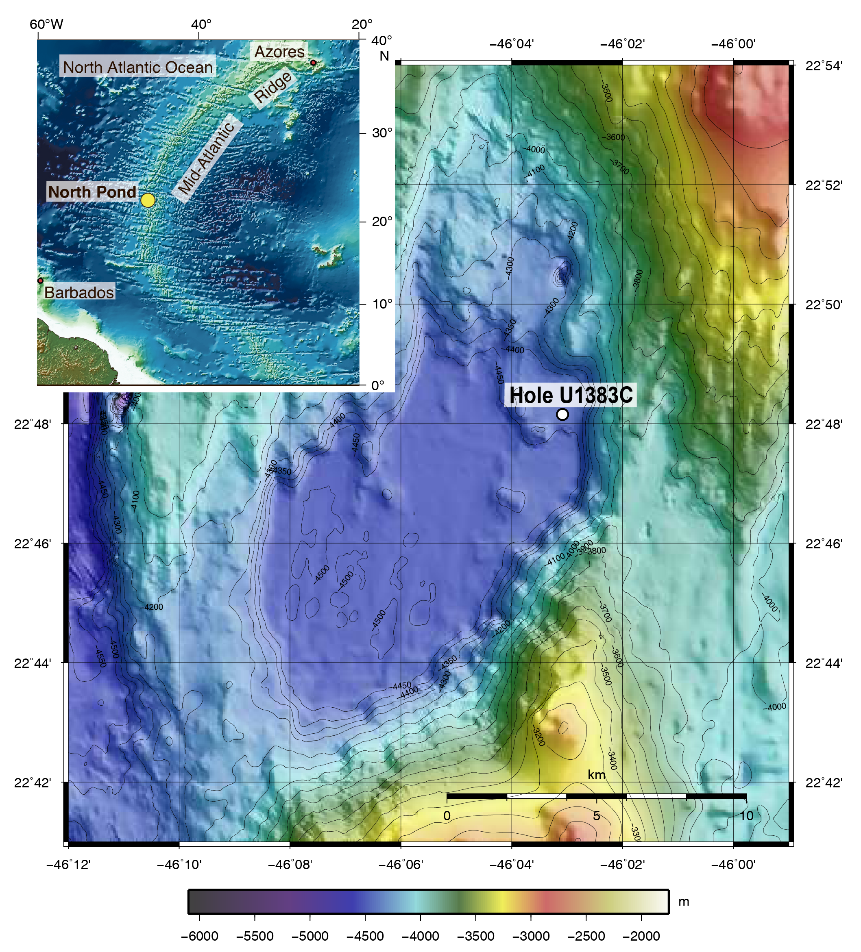


**Figure S1 | Location map and sampling sites.** The yellow dot in the upper left shows the location of North Pond on the western flank of Mid-Atlantic Ridge. The sampling sites are indicated with white dots in this study. The colored scale at the bottom indicates the water depth. The map is modified from the IODP Expedition 336 Preliminary Report ([Expedition 336 Scientists, 2012b](#_ENREF_7)).


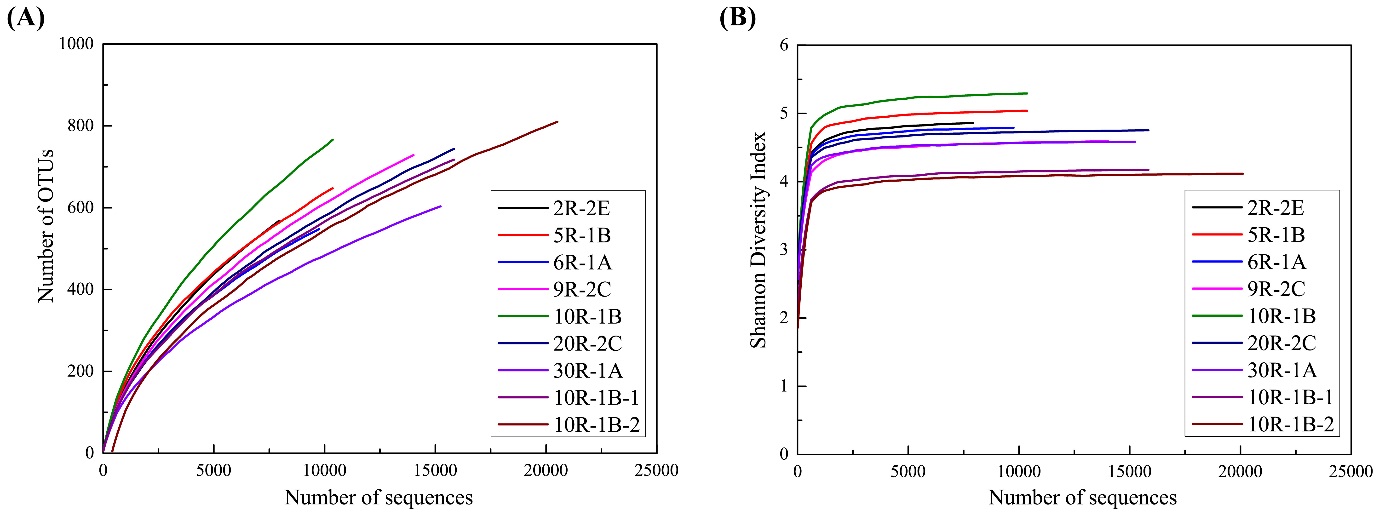


**Figure S2 | Alpha-diversity analysis of basaltic rock samples from U1383C.** (A) Rarefaction analysis. (B) Shannon Diversity Index curves.


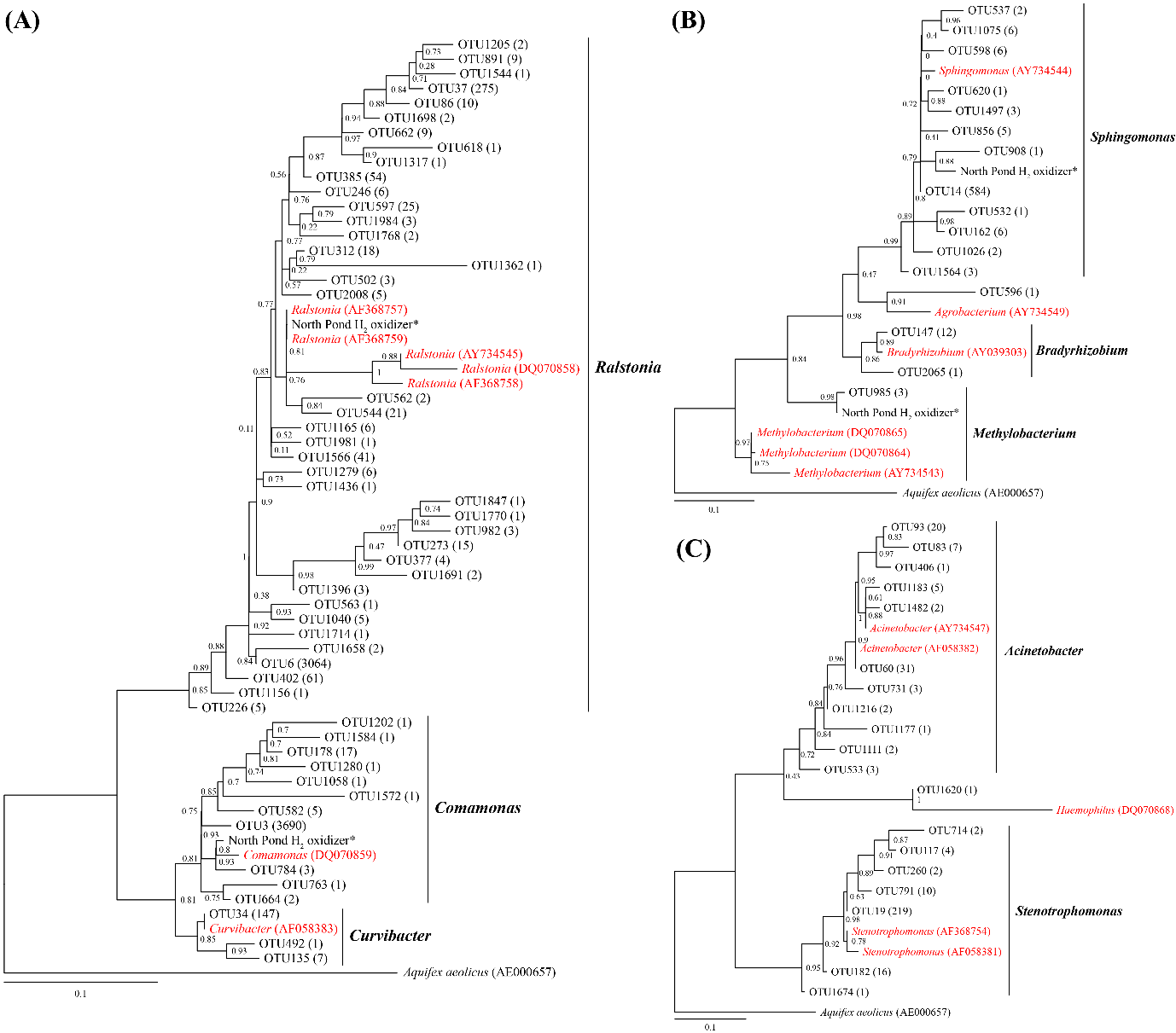


**Figure S3 | Comparative phylogenetic analysis of the suspected contaminating sequences in this study against sequences from the low-biomass contaminant database.** A representative set of sequences for each OTU are used due to the large number of sequences. (A) Betaproteobacterial related OTUs. (B) Alphaproteobacterial related OTUs. (C) Gammaproteobacterial related OTUs. Sequences from the low-biomass contaminant database are in red. The number in parentheses indicates total sequences of the designated OTU, including samples from 72, 97, 105, 137, 145, 221 and 304 mbsf. * indicates sequences from Hirayama et al. (2015). The 16S rRNA gene of *Aquifex aeolicus* (AE000657) is used as the outgroup. The scale bar indicates 0.1 nucleotide substitutions per site.


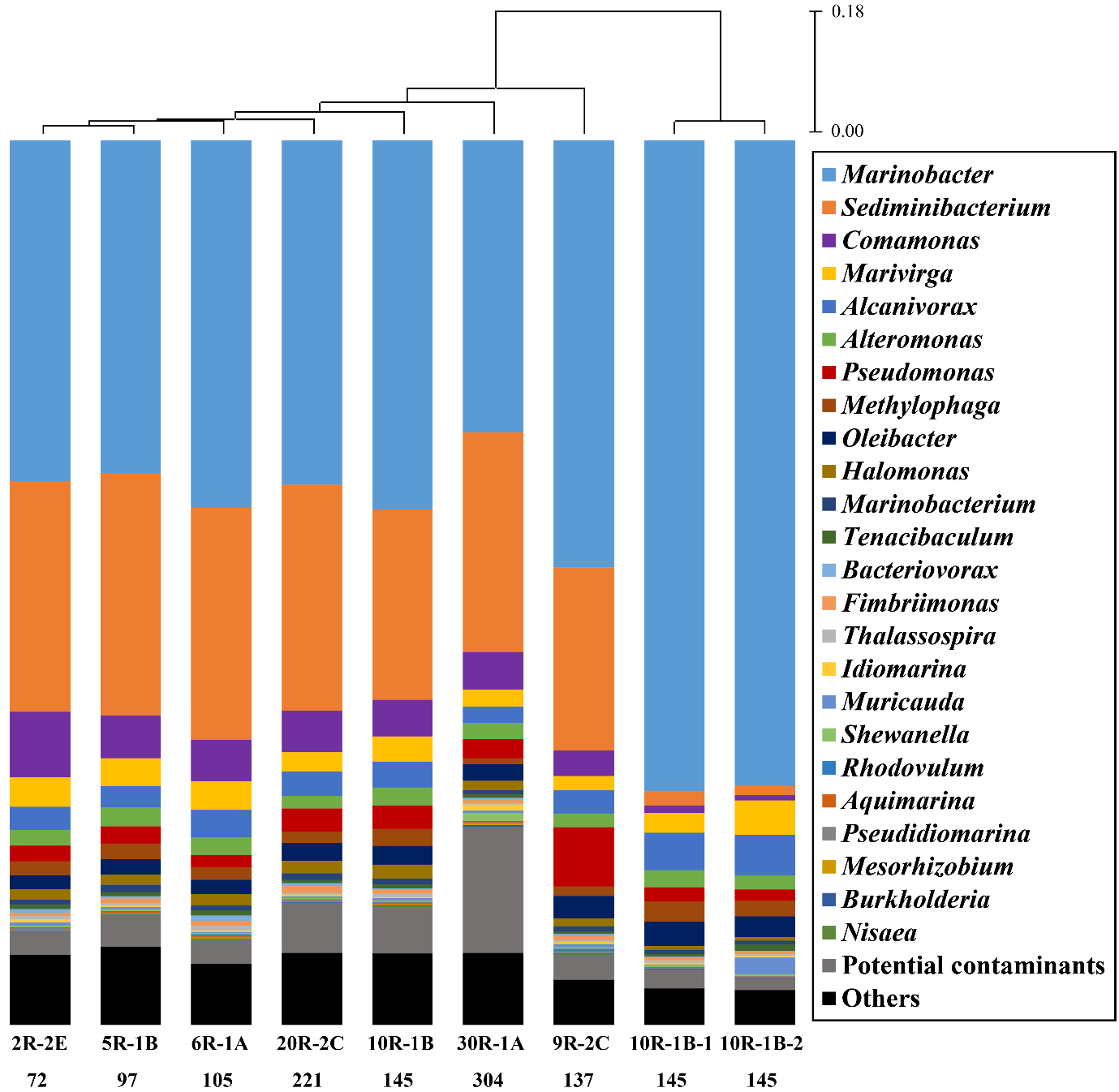


**Figure S4 | The bacterial community composition of basalt samples.** Colored bars indicate the percentage of the designated genus within each sample. Only genera with >0.1% abundance are listed. The remaining sequences are grouped to “Others”. The sequences identified as potential contaminants from commercial kits (including *Acinetobacter*, *Bradyrhizobium*, *Curvibacter*, *Ralstonia*, *Sphingomonas* and *Stenotrophomonas*) are grouped to "Potential contaminants". The hierarchical cluster dendrograms of microbial communities were based on a Bray-Curtis distance matrix. The scale bar indicates distance in length. The number indicates sample depth, mbsf.


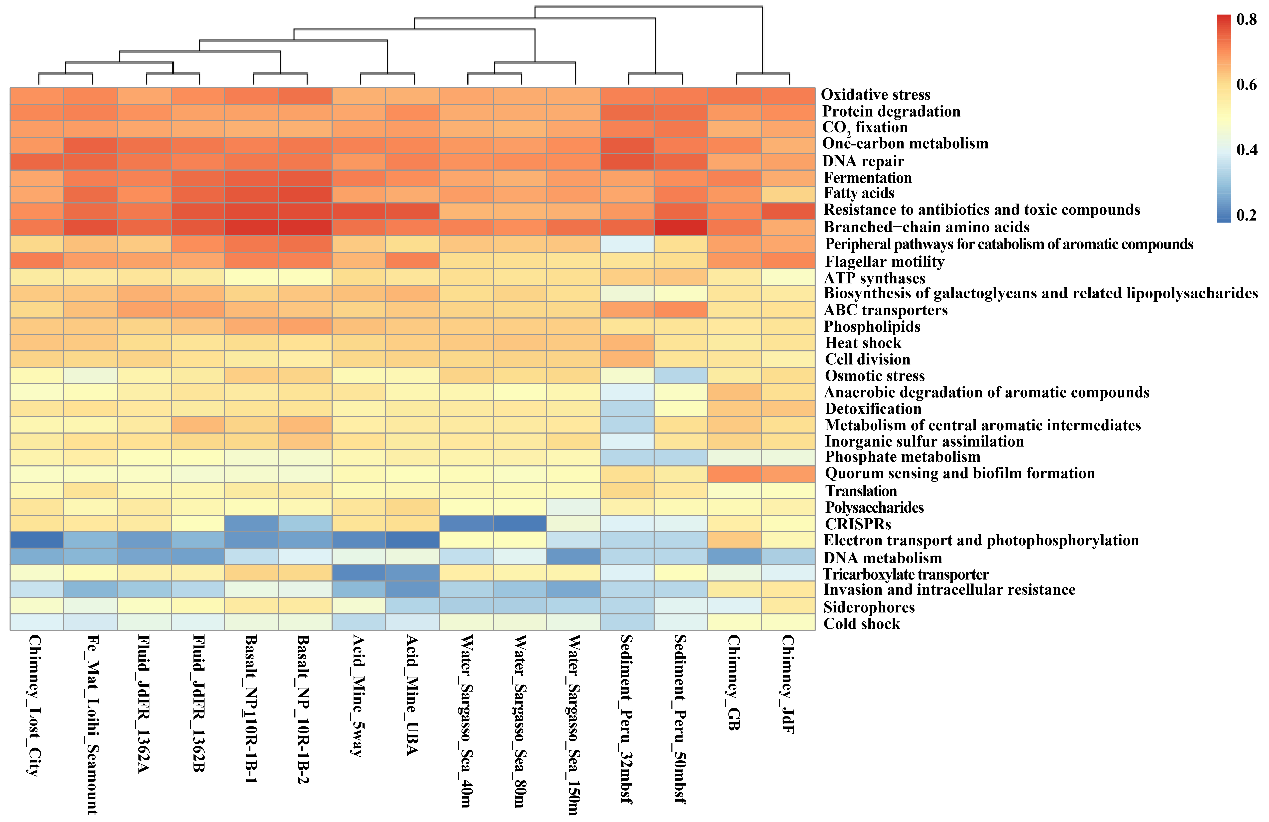


**Figure S5 | Hierarchical clustering based on Subsystems functional category with a maximum e-value of 10^-5^, a minimum identity of 30%.** The values in each category are normalized across the samples. The heatmap was clustered using complete clustering with a Bray-Curtis matrix and grouped at level 2 of the Subsystems annotation. Data calculated by MG-RAST ([Meyer et al., 2008](#_ENREF_19)).


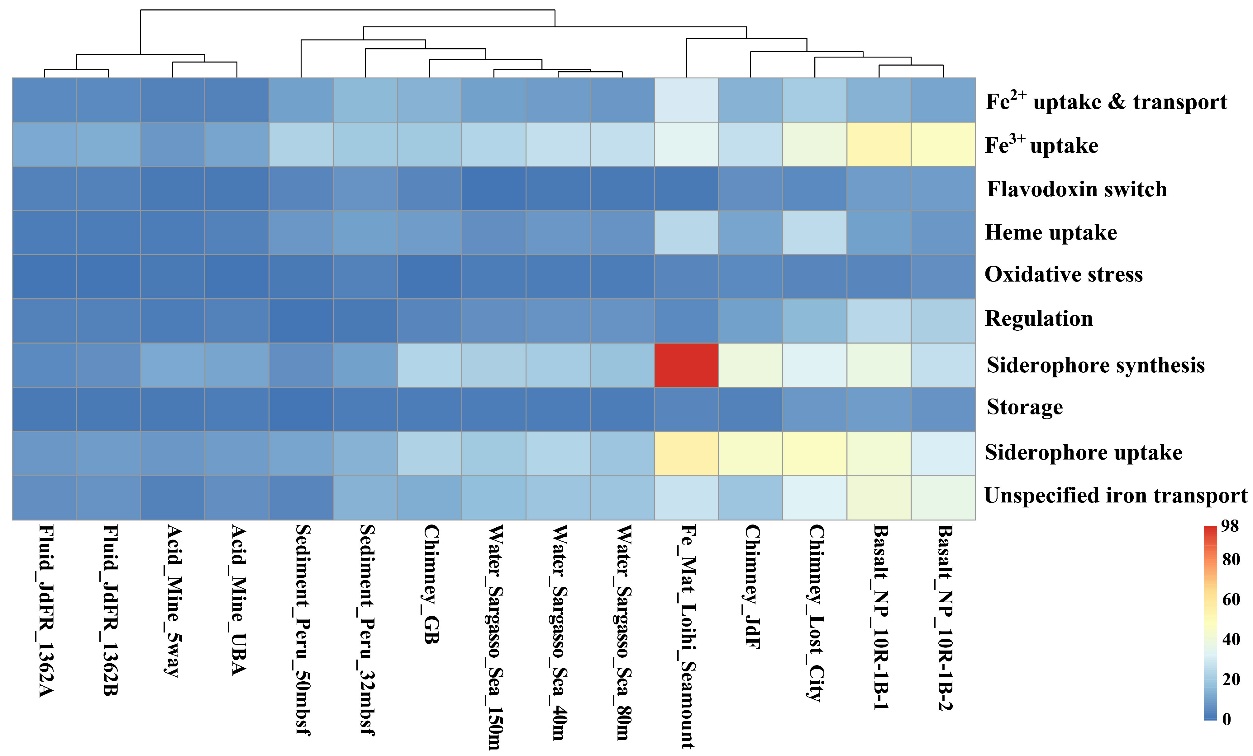


**Figure S6 | The relative abundance of iron uptake and transport pathway genes among metagenomes.** The colored bar on the right indicates the number of tBLASTn hits (e-value <10^-5^, identity >30% to the designated iron-related pathway) versus total reads.

**Table S1. Summary of microbiological studies in the oceanic crustal environments.**

| **Site** | **Rock type** | **Rock age** | **Major microbial groups** | **Cell density (cells cm^-3^)** | **Sample depth (mbsf^a^)** | **Water depth (m)** | **Temp. (°C)** | **Ref.** |
| --- | --- | --- | --- | --- | --- | --- | --- | --- |
| North Pond | aphyric/phyric basalt | ~8 Ma | γ-Proteobacteria, Bacteroidetes | Bd - 10^4*^  Bacteria | 70-324 | 4,425-4,492 | 5-25 | This study |
| Atlantis Massif | gabbro, peridotite | 1.5-2 Ma | α-, β-, γ-Proteobacteria | < 10^3*^  Bacteria | 62-1,391 | 1,645 | 14-102 | [I](#_ENREF_66) |
| Louisville Seamount | aphyric basalt,  hyaloclastite | 64-71 Ma | α-, β-, γ-Proteobacteria,  Actinobacteria, Bacteroidetes, Firmicutes | Bd - 10^4*^  Bacteria | 29-491 | 1,503-1,559 | NA | II |
| South Pacific Gyre | aphyric/phyric basalt | 13.5-100  Ma | NA | NA | 10-124 | 3,738- 5,708 | ~6.8 | III |
| Juan de Fuca Ridge  flank | crustal fluids from  subseafloor basalt | ~3.5 Ma | α-, δ-, γ-Proteobacteria,  Firmicutes, Bathyarchaeota  (MCG) | 10^3^ - 10^4*^  Varied | 370 | 2,667 | 65 | IV |
| Costa Rica Rift flank | crustal fluids from  subseafloor basalt | ~6.5 Ma | α-, γ-Proteobacteria,  Bacteroidetes, Planctomycetes, Verrucomicrobia | NA | 179-469 | 3,463 | ~60 | [V](#_ENREF_76) |
| Hawaii volcano | hyaloclastite | NA | Thaumarchaeota (MG I) | ~ 10^5#^  Archaea | 1,336-1,404 | 0 | 15 | VI |
| Loihi Seamount | basalt glass | 8 year -  >300 year | α-, γ-Proteobacteria,  Bacteroidetes | ~10^6&^  Bacteria | Se | 1,178-4,987 | ~2 | VII |
| East Pacific Rise | basalt glass | < 0.02 Ma | α-, γ-Proteobacteria | 10^6^ - 10^9&^  Bacteria | Se | 2,516-2,674 | ~2 | VIII |
| Knipovich & Mohns  Ridges | basalt glass | 20 year -  0.1 Ma | α-, γ-Proteobacteria,  Firmicutes | 10^5^ - 10^6&^  Bacteria | Se | 2,500-3,390 | -1 | IX |
| Ascension Fracture  Zone at Mid-Atlantic Ridge | basalt glass | NA | α-, β-, γ-Proteobacteria,  Actinobacteria, Firmicutes | NA | Se | 1,460-2,996 | 19.1-20.6 | X |

a: mbsf, meters below seafloor. Bd: below detection limit. NA: not applicable. Se: seafloor exposed.

Cell enumeration methods: * direct counting, # amino acid deduced, & quantitative PCR

I: Mason et al., 2010; II: Sylvan et al., 2013, Koppers et al., 2013; III: D'Hondt et al., 2013, Zhang et al., 2014; IV: [Jungbluth et al., 2013](#_ENREF_44), 2014, Lever et al., 2013, [Robador et al., 2015](#_ENREF_93); V: Nigro et al., 2012; VI: Fisk et al., 2003; VII: Templeton et al., 2005, Santelli et al., 2008, Jacobson Meyers et al., 2014; VIII: Santelli et al., 2008, 2009; IX: Lysnes et al., 2004, Einen et al., 2008; X: Rathsack et al., 2009.

**Table S2 | Bacterial and Archaeal primer sequences tested in this study.**

| **Primer name** | **Primer sequences (5’-3’)** | **Reference** |
| --- | --- | --- |
| 520F^1,3^ | XXXXXXXX- AYTGGGYDTAAAGNG | [Song et al. (2012](#_ENREF_27)) |
| 802R^1,3^ | TACNVGGGTATCTAATCC |  |
| U519F^2^ | YMGCCRCGGKAAHACC |  |
| Arch806R^2^ | CTACNSGGGTMTCTAAT |  |
| A2Fa^2^ | TTCCGGTTGATCCYGCCRGA | [Kim et al. (2011](#_ENREF_13)) |
| 519R^2^ | GWATTACCGCGGCKGCTG |  |
| Arch21F^2^ | TTCCGGTTGATCCYGCGGA | [DeLong (1992](#_ENREF_2)) |
| Arch958R^2^ | YCCGGCGTTGMTCCAATT |  |

1, bacterial primer; 2, archaeal primer; 3, primer for sequencing. X indicates the barcode sequence.

**Table S3 | ClustalW Alignment of the suspected contaminating sequences in this study against the sequences from the low-biomass contaminant database and sequences from Hirayama et al. (2015).**

| **Sequence ID from this study** | **Sequence from the contaminant database** | | | **Sequence from Hirayama et al. (2015)** | |
| --- | --- | --- | --- | --- | --- |
|  | Taxonomy | Accession No. | Identity  (%) | Isolation source | Identity  (%) |
| OTU34 (147) | *Curvibacter* | AF058383 | 100 | ND | ND |
| OTU60 (31) | *Acinetobacter* | AF058382 | 100 | ND | ND |
| OTU6 (3064) | *Ralstonia* | AF368757 | 99.1 | 1383C-2R-2D | 99.1 |
| OTU14 (584) | *Sphingomonas* | AY734544 | 98.7 | 1382A-6R-1A | 96.0 |
| OTU19 (219) | *Stenotrophomonas* | AF368754 | 98.7 | ND | ND |
| OTU147(12) | *Bradyrhizobium* | AY039303 | 98.2 | ND | ND |
| OTU784 (3) | *Comamonas* | DQ070859 | 96.9 | 1383C-30R-3C | 97.3 |
| OTU3 (3690) | *Comamonas* | DQ070859 | 96.0 | 1383C-30R-3C | 96.4 |
| OTU985 (3) | *Methylobacterium* | DQ070865 | 87.6 | 1382A-3R-4B | 99.1 |

The number in parentheses indicates total sequences of the designated OTU, including samples from 72, 97, 105, 137, 145, 221 and 304 mbsf. ND indicates not detected.

**Table S4 | Shared bacterial groups from selected basalts for Venn diagram.**

| **Basalt samples (mbsf)** | **Shared bacterial groups at class level** |
| --- | --- |
| 72, 105, 137, 145, 304 | Gammaproteobacteria, Sphingobacteria, Betaproteobacteria, Cytophagia, Alphaproteobacteria, Flavobacteria, Deltaproteobacteria, Fimbriimonadia, ML635J-21, Acidimicrobiia, Bacilli, Actinobacteria |
| 105, 137, 145, 304 | DA052 |
| 105, 137, 304 | Chlamydiia |
| 137, 145, 304 | Unclassified Proteobacteria |
| 145, 304 | Thermoleophilia, Deinococci, SJA-4 |
| 72 | Sphingobacteriia, 4C0d-2, Bacteroidia |
| 105 | Rubrobacteria, Chloroplast |
| 137 | Nostocophycideae |
| 145 | Acidobacteria-6 |
| 304 | C6, Phycisphaerae, BD7-11 |

**Table S5 | Spearman’s correlation of OTU abundance with depth.**

| **OTU ID** | **Taxonomical assignments** | | | **Sample depth (mbsf)** | | | | | | | **Spearman correlation**  **with depth** | |
| --- | --- | --- | --- | --- | --- | --- | --- | --- | --- | --- | --- | --- |
|  | **Class** | **Family** | **Genus** | **72** | **97** | **105** | **137** | **145** | **221** | **304** | **R value** | **P value** |
|  |  |  |  | **Relative abundance (%)** | | | | | | |  |  |
| OTU19* | Gammaproteobacteria | Xanthomonadaceae | *Stenotrophomonas* | 0.112 | 0.208 | 0.289 | 0.179 | 0.343 | 0.316 | 0.347 | 0.9 | 0.024 |
| OTU714* | Gammaproteobacteria | Xanthomonadaceae | *Stenotrophomonas* | 0.000 | 0.000 | 0.000 | 0.000 | 0.000 | 0.006 | 0.006 | 0.8 | 0.048 |
| OTU214 | Gammaproteobacteria | Pseudomonadaceae | *Pseudomonas* | 0.000 | 0.000 | 0.000 | 0.000 | 0.000 | 0.006 | 0.006 | 0.8 | 0.048 |
| OTU1835 | Gammaproteobacteria | Pseudomonadaceae | *Pseudomonas* | 0.012 | 0.009 | 0.000 | 0.000 | 0.000 | 0.000 | 0.000 | -0.8 | 0.048 |
| OTU783 | Gammaproteobacteria | Moraxellaceae | *Enhydrobacter* | 0.000 | 0.000 | 0.000 | 0.000 | 0.000 | 0.006 | 0.006 | 0.8 | 0.048 |
| OTU93* | Gammaproteobacteria | Moraxellaceae | *Acinetobacter* | 0.000 | 0.009 | 0.010 | 0.010 | 0.010 | 0.025 | 0.077 | 0.9 | 0.024 |
| OTU1836 | Gammaproteobacteria | Oleiphilaceae | NA | 0.000 | 0.000 | 0.000 | 0.000 | 0.000 | 0.006 | 0.006 | 0.8 | 0.048 |
| OTU258 | Gammaproteobacteria | Oceanospirillaceae | *Oleibacter* | 0.000 | 0.000 | 0.000 | 0.000 | 0.000 | 0.006 | 0.006 | 0.8 | 0.048 |
| OTU1577 | Gammaproteobacteria | Oceanospirillaceae | *Oleibacter* | 0.000 | 0.000 | 0.000 | 0.000 | 0.000 | 0.006 | 0.006 | 0.8 | 0.048 |
| OTU28 | Gammaproteobacteria | Oceanospirillaceae | *Oleibacter* | 0.037 | 0.094 | 0.109 | 0.457 | 0.172 | 0.403 | 0.405 | 0.8 | 0.048 |
| OTU1035 | Gammaproteobacteria | Oceanospirillaceae | *Oleibacter* | 0.000 | 0.028 | 0.020 | 0.070 | 0.029 | 0.081 | 0.039 | 0.8 | 0.048 |
| OTU849 | Gammaproteobacteria | Oceanospirillaceae | *Oleibacter* | 0.025 | 0.028 | 0.010 | 0.010 | 0.000 | 0.000 | 0.006 | -0.8 | 0.025 |
| OTU1645 | Gammaproteobacteria | Oceanospirillaceae | *Marinobacterium* | 0.000 | 0.000 | 0.000 | 0.000 | 0.000 | 0.006 | 0.006 | 0.8 | 0.048 |
| OTU827 | Gammaproteobacteria | Oceanospirillaceae | NA | 0.012 | 0.009 | 0.000 | 0.000 | 0.000 | 0.000 | 0.000 | -0.8 | 0.048 |
| OTU151 | Gammaproteobacteria | Halomonadaceae | *Halomonas* | 0.000 | 0.000 | 0.010 | 0.000 | 0.019 | 0.031 | 0.019 | 0.9 | 0.029 |
| OTU208 | Gammaproteobacteria | Alcanivoracaceae | *Alcanivorax* | 0.000 | 0.000 | 0.000 | 0.010 | 0.019 | 0.019 | 0.019 | 0.9 | 0.01 |
| OTU144 | Gammaproteobacteria | Alcanivoracaceae | *Alcanivorax* | 0.062 | 0.047 | 0.070 | 0.080 | 0.076 | 0.093 | 0.083 | 0.9 | 0.012 |
| OTU1256 | Gammaproteobacteria | Alcanivoracaceae | *Alcanivorax* | 0.000 | 0.000 | 0.000 | 0.000 | 0.000 | 0.006 | 0.013 | 0.8 | 0.048 |
| OTU1219 | Gammaproteobacteria | Alcanivoracaceae | *Alcanivorax* | 0.000 | 0.000 | 0.000 | 0.000 | 0.000 | 0.006 | 0.006 | 0.8 | 0.048 |
| OTU676 | Gammaproteobacteria | Alcanivoracaceae | *Alcanivorax* | 0.000 | 0.000 | 0.000 | 0.000 | 0.000 | 0.006 | 0.006 | 0.8 | 0.048 |
| OTU1209 | Gammaproteobacteria | Alcanivoracaceae | *Alcanivorax* | 0.000 | 0.000 | 0.000 | 0.000 | 0.000 | 0.006 | 0.006 | 0.8 | 0.048 |
| OTU143 | Gammaproteobacteria | Enterobacteriaceae | NA | 0.000 | 0.000 | 0.000 | 0.010 | 0.010 | 0.012 | 0.026 | 0.9 | 0.01 |
| OTU880 | Gammaproteobacteria | Shewanellaceae | *Shewanella* | 0.000 | 0.000 | 0.000 | 0.000 | 0.000 | 0.006 | 0.013 | 0.8 | 0.048 |
| OTU204 | Gammaproteobacteria | Idiomarinaceae | *Pseudidiomarina* | 0.000 | 0.000 | 0.000 | 0.000 | 0.000 | 0.006 | 0.006 | 0.8 | 0.048 |
| OTU713 | Gammaproteobacteria | Idiomarinaceae | *Pseudidiomarina* | 0.000 | 0.000 | 0.000 | 0.000 | 0.000 | 0.006 | 0.006 | 0.8 | 0.048 |
| OTU119 | Gammaproteobacteria | Alteromonadaceae | *Marinobacter* | 0.000 | 0.000 | 0.000 | 0.020 | 0.019 | 0.031 | 0.045 | 0.9 | 0.01 |
| OTU1172 | Gammaproteobacteria | Alteromonadaceae | *Marinobacter* | 0.000 | 0.000 | 0.000 | 0.000 | 0.010 | 0.019 | 0.013 | 0.9 | 0.029 |
| OTU1800 | Gammaproteobacteria | Alteromonadaceae | *Marinobacter* | 0.000 | 0.009 | 0.020 | 0.010 | 0.010 | 0.025 | 0.032 | 0.9 | 0.024 |
| OTU959 | Gammaproteobacteria | Alteromonadaceae | *Marinobacter* | 0.000 | 0.009 | 0.000 | 0.000 | 0.010 | 0.019 | 0.026 | 0.8 | 0.038 |
| OTU489 | Gammaproteobacteria | Alteromonadaceae | *Marinobacter* | 0.000 | 0.000 | 0.000 | 0.010 | 0.010 | 0.006 | 0.013 | 0.8 | 0.038 |
| OTU220 | Gammaproteobacteria | Alteromonadaceae | *Marinobacter* | 0.000 | 0.009 | 0.010 | 0.000 | 0.029 | 0.062 | 0.051 | 0.8 | 0.038 |
| OTU671 | Gammaproteobacteria | Alteromonadaceae | *Marinobacter* | 0.000 | 0.000 | 0.000 | 0.000 | 0.000 | 0.006 | 0.006 | 0.8 | 0.048 |
| OTU779 | Gammaproteobacteria | Alteromonadaceae | *Marinobacter* | 0.000 | 0.000 | 0.000 | 0.000 | 0.000 | 0.006 | 0.006 | 0.8 | 0.048 |
| OTU962 | Gammaproteobacteria | Alteromonadaceae | *Marinobacter* | 0.000 | 0.000 | 0.000 | 0.000 | 0.000 | 0.006 | 0.006 | 0.8 | 0.048 |
| OTU1723 | Gammaproteobacteria | Alteromonadaceae | *Marinobacter* | 0.000 | 0.000 | 0.000 | 0.000 | 0.000 | 0.006 | 0.006 | 0.8 | 0.048 |
| OTU309 | Gammaproteobacteria | Alteromonadaceae | *Marinobacter* | 0.000 | 0.000 | 0.000 | 0.000 | 0.000 | 0.006 | 0.006 | 0.8 | 0.048 |
| OTU1472 | Gammaproteobacteria | Alteromonadaceae | *Marinobacter* | 0.000 | 0.000 | 0.000 | 0.000 | 0.000 | 0.006 | 0.006 | 0.8 | 0.048 |
| OTU530 | Gammaproteobacteria | Alteromonadaceae | *Marinobacter* | 0.000 | 0.000 | 0.000 | 0.000 | 0.000 | 0.006 | 0.006 | 0.8 | 0.048 |
| OTU1778 | Gammaproteobacteria | Alteromonadaceae | *Marinobacter* | 0.000 | 0.000 | 0.000 | 0.000 | 0.000 | 0.006 | 0.006 | 0.8 | 0.048 |
| OTU1828 | Gammaproteobacteria | Alteromonadaceae | *Marinobacter* | 0.025 | 0.009 | 0.010 | 0.020 | 0.000 | 0.006 | 0.000 | -0.8 | 0.041 |
| OTU1595 | Gammaproteobacteria | Alteromonadaceae | *Marinobacter* | 0.037 | 0.009 | 0.000 | 0.000 | 0.000 | 0.000 | 0.000 | -0.8 | 0.048 |
| OTU464 | Gammaproteobacteria | Alteromonadaceae | *Marinobacter* | 0.012 | 0.009 | 0.000 | 0.000 | 0.000 | 0.000 | 0.000 | -0.8 | 0.048 |
| OTU187 | Gammaproteobacteria | Alteromonadaceae | *Marinobacter* | 0.012 | 0.009 | 0.000 | 0.000 | 0.000 | 0.000 | 0.000 | -0.8 | 0.048 |
| OTU1459 | Gammaproteobacteria | Alteromonadaceae | *Marinobacter* | 0.012 | 0.009 | 0.000 | 0.000 | 0.000 | 0.000 | 0.000 | -0.8 | 0.048 |
| OTU1653 | Gammaproteobacteria | Alteromonadaceae | *Marinobacter* | 0.025 | 0.028 | 0.020 | 0.020 | 0.019 | 0.006 | 0.013 | -0.9 | 0.007 |
| OTU547 | Gammaproteobacteria | Alteromonadaceae | *Alteromonas* | 0.000 | 0.000 | 0.000 | 0.040 | 0.019 | 0.031 | 0.051 | 0.9 | 0.029 |
| OTU1042 | Gammaproteobacteria | Alteromonadaceae | *Alteromonas* | 0.000 | 0.000 | 0.000 | 0.000 | 0.000 | 0.006 | 0.013 | 0.8 | 0.048 |
| OTU1020 | Gammaproteobacteria | Alteromonadaceae | *Alteromonas* | 0.000 | 0.000 | 0.000 | 0.000 | 0.000 | 0.006 | 0.006 | 0.8 | 0.048 |
| OTU1305 | Gammaproteobacteria | Alteromonadaceae | NA | 0.000 | 0.000 | 0.000 | 0.000 | 0.000 | 0.006 | 0.019 | 0.8 | 0.048 |
| OTU1233 | Gammaproteobacteria | Alteromonadaceae | NA | 0.000 | 0.000 | 0.000 | 0.000 | 0.000 | 0.006 | 0.013 | 0.8 | 0.048 |
| OTU1347 | Gammaproteobacteria | NA | NA | 0.000 | 0.000 | 0.000 | 0.000 | 0.000 | 0.006 | 0.006 | 0.8 | 0.048 |
| OTU66 | Deltaproteobacteria | 0319-6G20 | NA | 0.000 | 0.000 | 0.000 | 0.010 | 0.010 | 0.050 | 0.026 | 0.9 | 0.014 |
| OTU1037 | Deltaproteobacteria | NA | NA | 0.000 | 0.000 | 0.000 | 0.000 | 0.000 | 0.006 | 0.006 | 0.8 | 0.048 |
| OTU55 | Betaproteobacteria | Procabacteriaceae | NA | 0.012 | 0.019 | 0.010 | 0.030 | 0.019 | 0.056 | 0.058 | 0.9 | 0.024 |
| OTU1260 | Betaproteobacteria | NA | NA | 0.000 | 0.000 | 0.000 | 0.000 | 0.000 | 0.006 | 0.013 | 0.8 | 0.048 |
| OTU1279* | Betaproteobacteria | Oxalobacteraceae | *Ralstonia* | 0.000 | 0.000 | 0.000 | 0.000 | 0.010 | 0.006 | 0.026 | 0.9 | 0.029 |
| OTU6* | Betaproteobacteria | Oxalobacteraceae | *Ralstonia* | 1.269 | 1.615 | 1.413 | 1.283 | 2.841 | 3.374 | 10.775 | 0.9 | 0.024 |
| OTU402* | Betaproteobacteria | Oxalobacteraceae | *Ralstonia* | 0.025 | 0.066 | 0.020 | 0.040 | 0.067 | 0.068 | 0.180 | 0.8 | 0.034 |
| OTU1165* | Betaproteobacteria | Oxalobacteraceae | *Ralstonia* | 0.000 | 0.009 | 0.000 | 0.000 | 0.010 | 0.012 | 0.013 | 0.8 | 0.038 |
| OTU1396* | Betaproteobacteria | Oxalobacteraceae | *Ralstonia* | 0.000 | 0.000 | 0.000 | 0.000 | 0.000 | 0.006 | 0.013 | 0.8 | 0.048 |
| OTU711 | Betaproteobacteria | Oxalobacteraceae | NA | 0.000 | 0.000 | 0.000 | 0.000 | 0.000 | 0.012 | 0.013 | 0.8 | 0.048 |
| OTU311 | Betaproteobacteria | Oxalobacteraceae | NA | 0.000 | 0.000 | 0.000 | 0.000 | 0.000 | 0.006 | 0.006 | 0.8 | 0.048 |
| OTU759 | Betaproteobacteria | Oxalobacteraceae | NA | 0.000 | 0.000 | 0.000 | 0.000 | 0.000 | 0.006 | 0.006 | 0.8 | 0.048 |
| OTU991 | Betaproteobacteria | Comamonadaceae | *Methylibium* | 0.000 | 0.000 | 0.000 | 0.000 | 0.000 | 0.012 | 0.026 | 0.8 | 0.048 |
| OTU178 | Betaproteobacteria | Comamonadaceae | *Comamonas* | 0.087 | 0.019 | 0.020 | 0.020 | 0.010 | 0.019 | 0.000 | -0.9 | 0.024 |
| OTU666 | Betaproteobacteria | Comamonadaceae | NA | 0.000 | 0.000 | 0.000 | 0.000 | 0.010 | 0.006 | 0.013 | 0.9 | 0.029 |
| OTU884 | Betaproteobacteria | Comamonadaceae | NA | 0.000 | 0.000 | 0.000 | 0.000 | 0.000 | 0.006 | 0.006 | 0.8 | 0.048 |
| OTU308 | Betaproteobacteria | Comamonadaceae | NA | 0.000 | 0.000 | 0.000 | 0.000 | 0.000 | 0.006 | 0.006 | 0.8 | 0.048 |
| OTU87 | Betaproteobacteria | Comamonadaceae | NA | 0.075 | 0.047 | 0.060 | 0.060 | 0.048 | 0.031 | 0.026 | -0.8 | 0.048 |
| OTU41 | Betaproteobacteria | Burkholderiaceae | *Burkholderia* | 0.012 | 0.038 | 0.030 | 0.070 | 0.076 | 0.087 | 0.218 | 1 | 0.003 |
| OTU352 | Betaproteobacteria | Burkholderiaceae | *Burkholderia* | 0.000 | 0.000 | 0.000 | 0.000 | 0.000 | 0.006 | 0.006 | 0.8 | 0.048 |
| OTU421 | Betaproteobacteria | NA | NA | 0.000 | 0.000 | 0.000 | 0.000 | 0.010 | 0.006 | 0.013 | 0.9 | 0.029 |
| OTU501 | Betaproteobacteria | NA | NA | 0.000 | 0.000 | 0.000 | 0.000 | 0.000 | 0.006 | 0.013 | 0.8 | 0.048 |
| OTU1746 | Betaproteobacteria | NA | NA | 0.012 | 0.009 | 0.000 | 0.000 | 0.000 | 0.000 | 0.000 | -0.8 | 0.048 |
| OTU206 | Alphaproteobacteria | Rhodobacteraceae | NA | 0.037 | 0.019 | 0.000 | 0.000 | 0.000 | 0.000 | 0.000 | -0.8 | 0.048 |
| OTU1262 | Alphaproteobacteria | Rhodobacteraceae | NA | 0.012 | 0.009 | 0.000 | 0.000 | 0.000 | 0.000 | 0.000 | -0.8 | 0.048 |
| OTU215 | Alphaproteobacteria | NA | NA | 0.000 | 0.000 | 0.000 | 0.010 | 0.010 | 0.006 | 0.013 | 0.8 | 0.038 |
| OTU1101 | Alphaproteobacteria | NA | NA | 0.000 | 0.000 | 0.000 | 0.000 | 0.000 | 0.006 | 0.006 | 0.8 | 0.048 |
| OTU33 | Alphaproteobacteria | NA | NA | 0.112 | 0.104 | 0.070 | 0.268 | 0.143 | 0.515 | 0.449 | 0.8 | 0.048 |
| OTU914 | Bacilli | Bacillaceae | *Bacillus* | 0.000 | 0.000 | 0.000 | 0.000 | 0.000 | 0.006 | 0.006 | 0.8 | 0.048 |
| OTU188 | Flavobacteriia | Flavobacteriaceae | *Tenacibaculum* | 0.062 | 0.047 | 0.040 | 0.010 | 0.019 | 0.006 | 0.006 | -0.9 | 0.007 |
| OTU45 | Flavobacteriia | Flavobacteriaceae | NA | 0.087 | 0.066 | 0.080 | 0.129 | 0.095 | 0.149 | 0.154 | 0.9 | 0.024 |
| OTU440 | Flavobacteriia | Flavobacteriaceae | NA | 0.000 | 0.000 | 0.000 | 0.000 | 0.000 | 0.006 | 0.006 | 0.8 | 0.048 |
| OTU1261 | Flavobacteriia | Flavobacteriaceae | NA | 0.000 | 0.000 | 0.000 | 0.000 | 0.000 | 0.006 | 0.006 | 0.8 | 0.048 |
| OTU21 | Cytophagia | Flammeovirgaceae | *Ekhidna* | 1.119 | 0.860 | 1.055 | 0.736 | 0.820 | 0.732 | 0.758 | -0.8 | 0.048 |
| OTU761 | Cytophagia | Flammeovirgaceae | NA | 0.012 | 0.009 | 0.000 | 0.000 | 0.000 | 0.000 | 0.000 | -0.8 | 0.048 |
| OTU57 | Cytophagia | Flammeovirgaceae | NA | 0.112 | 0.057 | 0.050 | 0.040 | 0.048 | 0.025 | 0.039 | -0.9 | 0.007 |
| OTU493 | Cytophagia | Flammeovirgaceae | NA | 0.012 | 0.019 | 0.010 | 0.010 | 0.010 | 0.000 | 0.006 | -0.9 | 0.007 |
| OTU1594 | Sphingobacteria | Chitinophagaceae | *Sediminibacterium* | 0.000 | 0.000 | 0.000 | 0.000 | 0.010 | 0.006 | 0.019 | 0.9 | 0.029 |
| OTU1563 | Sphingobacteria | Chitinophagaceae | *Sediminibacterium* | 0.000 | 0.000 | 0.000 | 0.000 | 0.010 | 0.006 | 0.013 | 0.9 | 0.029 |
| OTU1514 | Sphingobacteria | Chitinophagaceae | *Sediminibacterium* | 0.000 | 0.000 | 0.000 | 0.000 | 0.000 | 0.006 | 0.019 | 0.8 | 0.048 |
| OTU503 | Sphingobacteria | Chitinophagaceae | *Sediminibacterium* | 0.025 | 0.038 | 0.030 | 0.010 | 0.019 | 0.019 | 0.006 | -0.8 | 0.048 |
| OTU338 | Sphingobacteria | Chitinophagaceae | *Sediminibacterium* | 0.037 | 0.047 | 0.060 | 0.010 | 0.010 | 0.000 | 0.000 | -0.8 | 0.025 |
| OTU1105 | Sphingobacteria | Chitinophagaceae | *Sediminibacterium* | 0.211 | 0.151 | 0.179 | 0.099 | 0.114 | 0.105 | 0.051 | -0.9 | 0.024 |
| OTU799 | Sphingobacteria | Chitinophagaceae | *Sediminibacterium* | 0.062 | 0.085 | 0.050 | 0.030 | 0.029 | 0.031 | 0.013 | -0.9 | 0.024 |
| OTU702 | Sphingobacteria | Chitinophagaceae | *Sediminibacterium* | 0.062 | 0.085 | 0.070 | 0.060 | 0.057 | 0.050 | 0.039 | -0.9 | 0.012 |
| OTU347 | Fimbriimonadia | Fimbriimonadaceae | *Fimbriimonas* | 0.000 | 0.000 | 0.000 | 0.000 | 0.000 | 0.006 | 0.006 | 0.8 | 0.048 |
| OTU341 | Actinobacteria | Corynebacteriaceae | *Corynebacterium* | 0.000 | 0.000 | 0.000 | 0.000 | 0.010 | 0.012 | 0.013 | 0.9 | 0.01 |
| OTU76 | DA052 | NA | NA | 0.000 | 0.009 | 0.010 | 0.010 | 0.010 | 0.025 | 0.032 | 0.9 | 0.024 |

* indicates potential contaminating sequence. NA indicates not assigned to taxon.

**Table S6 | Summary of sequences from the basalt metagenomes.**

| **Properties** | **10R-1B-1** | **10R-1B-2** |
| --- | --- | --- |
| Clean reads | 30,940,000 | 31,030,000 |
| Average read length (bp) | 100 | 100 |
| Contigs (length >500 bp) | 11,114 | 6,934 |
| Average contig length (bp) | 1,731 | 2,010 |
| Contigs GC content (%) | 54.2 | 53.0 |
| Predicted genes | 21,842 | 15,155 |
| Bacterial derived gene (%) | 99.3 | 99.4 |
| Archaeal derived gene (%) | 0.2 | 0.2 |

**References:**

D'Hondt, S., Inagaki, F., Alvarez Zarikian, C., and the, I.E.S.P. (2013). IODP Expedition 329: Life and Habitability Beneath the Seafloor of the South Pacific Gyre. *Sci. Dril.* 15**,** 4-10. doi: 10.5194/sd-15-4-2013.

DeLong, E.F. (1992). Archaea in coastal marine environments. *Proc. Natl. Acad. Sci. U.S.A.* 89**,** 5685-5689. doi: 10.1073/pnas.89.12.5685.

Einen, J., Thorseth, I.H., and Ovreås, L. (2008). Enumeration of Archaea and Bacteria in seafloor basalt using real-time quantitative PCR and fluorescence microscopy. *FEMS Microbiol. Lett.* 282**,** 182-187. doi: 10.1111/j.1574-6968.2008.01119.x.

Expedition 327 Scientists (2011). Methods. *In* Fisher, A.T., Tsuji, T., Petronotis, K., and the Expedition 327 Scientists, *Proc. IODP,* **327**: Tokyo (Integrated Ocean Drilling Program Management International, Inc.). doi:10.2204/iodp.proc.327.102.2011.

Expedition 330 Scientists (2012). Methods. *In* Koppers, A.A.P., Yamazaki, T., Geldmacher, J., and the Expedition 330 Scientists, *Proc. IODP*, 330: Tokyo (Integrated Ocean Drilling Program Management International, Inc.). doi:10.2204/iodp.proc.330.102.2012.

Expedition 336 Scientists (2012a). Methods in *Proc. IODP, 336*. (eds Edwards KJ, Bach W, Klaus A, and the Expedition 336 Scientists) (Integrated Ocean Drilling Program Management International, Inc.). doi: 10.2204/iodp.proc.336.102.2012.

Expedition 336 Scientists (2012b). Mid-Atlantic Ridge microbiology: Initation of long-term coupled microbiological, geochemical, and hydrological experimentation within the seaﬂoor at North Pond, western ﬂank of the Mid-Atlantic Ridge. *IODP Prel. Rep.* 336. doi: 10.2204/iodp.pr.336.2012.

Fisk, M.R., Storrie-Lombardi, M.C., Douglas, S., Popa, R., McDonald, G., and Di Meo-Savoie, C. (2003). Evidence of biological activity in Hawaiian subsurface basalts. *Geochem. Geophys. Geosyst.* 4**,** 1103. doi: 10.1029/2002gc000387.

Jacobson Meyers, M.E., Sylvan, J.B., and Edwards, K.J. (2014). Extracellular Enzyme Activity and Microbial Diversity Measured on Seafloor Exposed Basalts from Loihi Seamount Indicate the Importance of Basalts to Global Biogeochemical Cycling. *Appl. Environ. Microbiol.* 80**,** 4854-4864. doi: 10.1128/aem.01038-14.

Jungbluth, S.P., Grote, J., Lin, H.T., Cowen, J.P., and Rappe, M.S. (2013). Microbial diversity within basement fluids of the sediment-buried Juan de Fuca Ridge flank. *ISME J.* 7**,** 161-172. doi: 10.1038/ismej.2012.73.

Jungbluth, S.P., Lin, H.T., Cowen, J.P., Glazer, B.T., and Rappe, M.S. (2014). Phylogenetic diversity of microorganisms in subseafloor crustal fluids from Holes 1025C and 1026B along the Juan de Fuca Ridge flank. *Front. Microbiol.* 5**,** 119. doi: 10.3389/fmicb.2014.00119.

Kallmeyer, J., Smith, D.C., Spivack, A.J., and D'Hondt, S. (2008). New cell extraction procedure applied to deep subsurface sediments. *Limnol. Oceanogr. Methods* 6**,** 236-245. doi: 10.4319/lom.2008.6.236.

Kim, M., Morrison, M., and Yu, Z.T. (2011). Evaluation of different partial 16S rRNA gene sequence regions for phylogenetic analysis of microbiomes. *J. Microbiol. Methods* 84**,** 81-87. doi: 10.1016/j.mimet.2010.10.020.

Koppers, A.A.P., Yamazaki, T., Geldmacher, J., and the, I.E.S.P. (2013). IODP Expedition 330: Drilling the Louisville Seamount Trail in the SW Pacific. *Sci. Dril.* 15**,** 11-22. doi: 10.5194/sd-15-11-2013.

Lever, M.A., Alperin, M., Engelen, B., Inagaki, F., Nakagawa, S., Steinsbu, B.O., Teske, A., and Sci, I.E. (2006). Trends in basalt and sediment core contamination during IODP Expedition 301. *Geomicrobiol. J.* 23**,** 517-530. doi: 10.1080/01490450600897245.

Lever, M.A., Rouxel, O., Alt, J.C., Shimizu, N., Ono, S., Coggon, R.M., Shanks, W.C., 3rd, Lapham, L., Elvert, M., Prieto-Mollar, X., Hinrichs, K.U., Inagaki, F., and Teske, A. (2013). Evidence for microbial carbon and sulfur cycling in deeply buried ridge flank basalt. *Science* 339**,** 1305-1308. doi: 10.1126/science.1229240.

Lysnes, K., Thorseth, I.H., Steinsbu, B.O., Ovreas, L., Torsvik, T., and Pedersen, R.B. (2004). Microbial community diversity in seafloor basalt from the Arctic spreading ridges. *FEMS Microbiol. Ecol.* 50**,** 213-230. doi: 10.1016/j.femsec.2004.06.014.

Mason, O.U., Nakagawa, T., Rosner, M., Van Nostrand, J.D., Zhou, J., Maruyama, A., Fisk, M.R., and Giovannoni, S.J. (2010). First investigation of the microbiology of the deepest layer of ocean crust. *PLoS ONE* 5**,** e15399. doi: 10.1371/journal.pone.0015399.

Meyer, F., Paarmann, D., D'Souza, M., Olson, R., Glass, E.M., Kubal, M., Paczian, T., Rodriguez, A., Stevens, R., Wilke, A., Wilkening, J., and Edwards, R.A. (2008). The metagenomics RAST server - a public resource for the automatic phylogenetic and functional analysis of metagenomes. *BMC Bioinformatics* 9. doi: 10.1186/1471-2105-9-386.

Nigro, L.M., Harris, K., Orcutt, B.N., Hyde, A., Clayton-Luce, S., Becker, K., and Teske, A. (2012). Microbial communities at the borehole observatory on the Costa Rica Rift flank (Ocean Drilling Program Hole 896A). *Front. Microbiol.* 3**,** 232. doi: 10.3389/fmicb.2012.00232.

Rathsack, K., Stackebrandt, E., Reitner, J., and Schumann, G. (2009). Microorganisms Isolated from Deep Sea Low-temperature Influenced Oceanic Crust Basalts and Sediment Samples Collected along the Mid-Atlantic Ridge. *Geomicrobiol. J.* 26**,** 264-274. doi: 10.1080/01490450902892456.

Robador, A., Jungbluth, S.P., LaRowe, D., Bowers, R., Rappe, M., Amend, J., and Cowen, J. (2015). Activity and phylogenetic diversity of sulfate-reducing microorganisms in low-temperature subsurface fluids within the upper oceanic crust. *Front. Microbiol.* 5**,** 748. doi: 10.3389/fmicb.2014.00748.

Santelli, C.M., Edgcomb, V.P., Bach, W., and Edwards, K.J. (2009). The diversity and abundance of bacteria inhabiting seafloor lavas positively correlate with rock alteration. *Environ. Microbiol.* 11**,** 86-98. doi: 10.1111/j.1462-2920.2008.01743.x.

Santelli, C.M., Orcutt, B.N., Banning, E., Bach, W., Moyer, C.L., Sogin, M.L., Staudigel, H., and Edwards, K.J. (2008). Abundance and diversity of microbial life in ocean crust. *Nature* 453**,** 653-656. doi: 10.1038/nature06899.

Smith, A., Popa, R., Fisk, M., Nielsen, M., Wheat, C.G., Jannasch, H.W., Fisher, A.T., Becker, K., Sievert, S.M., and Flores, G. (2011). In situ enrichment of ocean crust microbes on igneous minerals and glasses using an osmotic flow-through device. *Geochem. Geophys. Geosyst.* 12. doi: 10.1029/2010gc003424.

Smith, D.C., Spivack, A.J., Fisk, M.R., Haveman, S.A., Staudigel, H., and and the Leg 185 Shipboard Scientific Party (2000). Methods for quantifying potential microbial contamination during deep ocean coring. *ODP Tech. Note*, 28. doi:10.2973/odp.tn.28.2000.

Song, Z.Q., Wang, F.P., Zhi, X.Y., Chen, J.Q., Zhou, E.M., Liang, F., Xiao, X., Tang, S.K., Jiang, H.C., Zhang, C.L., Dong, H., and Li, W.J. (2012). Bacterial and archaeal diversities in Yunnan and Tibetan hot springs, China. *Environ. Microbiol.* 15**,** 1160-1175. doi: 10.1111/1462-2920.12025.

Sylvan, J.B., Morono, Y., Grim, S.L., Inagaki, F., and Edwards, K.J. (2013). Deep subsurface microbiology of 64-71 million year old inactive seamounts along the Louisville Seamount Chain. Abstract B22B-05 presented at 2013 Fall Meeting, AGU, San Francisco, Calif., 9-13 Dec. doi: <http://abstractsearch.agu.org/meetings/2013/FM/B22B-05.html>.

Templeton, A.S., Staudigel, H., and Tebo, B.M. (2005). Diverse Mn(II)-oxidizing bacteria isolated from submarine basalts at Loihi seamount. *Geomicrobiol. J.* 22**,** 127-139. doi: 10.1080/01490450590945951.

Zhang, G.-L., and Smith-Duque, C. (2014). Seafloor basalt alteration and chemical change in the ultra thinly sedimented South Pacific. *Geochem. Geophys. Geosyst.* 15**,** 3066-3080. doi: 10.1002/2013gc005141.
